# The Endometrial Transcription Landscape of MRKH Syndrome

**DOI:** 10.3389/fcell.2020.572281

**Published:** 2020-09-24

**Authors:** Thomas Hentrich, André Koch, Nico Weber, Alexander Kilzheimer, Ana Maia, Simone Burkhardt, Katharina Rall, Nicolas Casadei, Oliver Kohlbacher, Olaf Riess, Julia Maria Schulze-Hentrich, Sara Yvonne Brucker

**Affiliations:** ^1^Institute of Medical Genetics and Applied Genomics, University of Tübingen, Tübingen, Germany; ^2^Department of Obstetrics and Gynecology, University of Tübingen, Tübingen, Germany; ^3^Applied Bioinformatics, Department of Computer Science, University of Tübingen, Tübingen, Germany; ^4^Institute for Bioinformatics and Medical Informatics, University of Tübingen, Tübingen, Germany; ^5^Division of Molecular Genome Analysis, German Cancer Research Center (DKFZ), Heidelberg, Germany; ^6^Centre for Rare Diseases, University of Tübingen, Tübingen, Germany; ^7^NGS Competence Center Tübingen (NCCT), Tübingen, Germany; ^8^Institute for Translational Bioinformatics, University Hospital Tübingen, Tübingen, Germany; ^9^Biomolecular Interactions, Max Planck Institute for Developmental Biology, Tübingen, Germany

**Keywords:** endometrium, uterus, MRKH, rare disease, transcriptome

## Abstract

The Mayer-Rokitansky-Küster-Hauser (MRKH) syndrome (OMIM 277000) is characterized by agenesis of the uterus and upper part of the vagina in females with normal ovarian function. While genetic causes have been identified for a small subset of patients and epigenetic mechanisms presumably contribute to the pathogenic unfolding, too, the etiology of the syndrome has remained largely enigmatic. A comprehensive understanding of gene activity in the context of the disease is crucial to identify etiological components and their potential interplay. So far, this understanding is lacking, primarily due to the scarcity of samples and suitable tissue. In order to close this gap, we profiled endometrial tissue of uterus rudiments in a large cohort of MRKH patients using RNA-seq and thereby provide a genome-wide view on the altered transcription landscape of the MRKH syndrome. Differential and co-expression analyses of the data identified cellular processes and candidate genes that converge on a core network of interconnected regulators that emerge as pivotal for the perturbed expression space. With these results and browsable access to the rich data through an online tool we seek to accelerate research to unravel the underlying biology of the syndrome.

## Introduction

Mayer-Rokitansky-Küster-Hauser (MRKH) syndrome (OMIM 277000) is the second most common cause of primary amenorrhea with an incidence rate of about one in 4000 to 5000 female births (Herlin et al., 2016). It is defined by agenesis of the uterus and the upper part of the vagina in 46,XX females with normal ovarian function and normal secondary sexual characteristics. The syndrome may occur either in an isolated form (type 1) or in association with extragenital abnormalities (type 2) such as renal or skeletal malformations (Oppelt et al., 2012; Rall et al., 2015).

The spectrum of malformation encountered in MRKH patients suggests the disease to originate from a developmental defect of the intermediate mesoderm during embryogenesis, yet the etiology of the syndrome remains largely enigmatic. While most cases are sporadic, familial cases exist and imply a genetic component in the etiology (Nik-Zainal et al., 2011; Herlin et al., 2014; Tewes et al., 2015). Specifically, chromosomal aberrations in 1q21.1, 16p11.2, 17q12, and 22q11 as well as mutations in *LHX1*, *TBX6*, *RBM8A*, and *WNT9B* have been linked to MRKH. Additionally, mutations of *WNT4* cause an atypical form of the syndrome characterized by hyperandrogenism (Ledig and Wieacker, 2018).

*LHX1*, *WNT4*, and *WNT9B* play important roles in the formation of the Müllerian Ducts (MD) from the coelomic epithelium in gestational week six (Ledig et al., 2012; Mullen and Behringer, 2014). The freshly formed MDs start growing caudally along the Wolffian Ducts. By week eight, both MDs begin to fuse and make contact with the uterovaginal sinus. In males, the MDs start to regress after week ten under the influence of *AMH* and *WNT7A*. In females, however, they differentiate into ovaries, uterus, cervix, and vagina under control of *ESR1*, *HOXA*, and *WNT* genes. In this context, *HOXA9*, *HOXA10*, *HOXA11*, and *HOXA13* are essential for correct tissue patterning. Their expression is tightly controlled through *Wnt signaling* and histone methylation marks (Robboy et al., 2017; Roly et al., 2018; Jambhekar et al., 2019) suggesting epigenetic principles to also play a role in the unfolding of the disease.

Toward a better understanding of the etiology, examining perturbed gene activity on a genome-wide scale promises to identify regulatory hubs on which genetic or epigenetic contributions converge. Attempts to identify the molecular mechanisms of the syndrome have been hampered by the lack of a comprehensive transcriptome profile for primary tissue in MRKH patients. This obstacle can partly be attributed to the fact that patients do not always have uterus rudiments with a complete endometrial layer and to the scarcity of uterine tissue resulting from challenging collection and biobanking efforts.

In order to close this gap, we have assembled a large and unique cohort of MRKH type 1 and 2 patients and profiled the transcriptome in endometrial tissue. The expression landscape that emerged along comprehensive differential and co-expression analyses of these data mapped known and novel candidate genes and identified regulatory networks that seemingly drive the underlying disease biology. By offering an online tool that allows navigating and downloading these rich data from single genes to pathways, we seek to provide a much-needed building block for the research community to understand the molecular pathomechanisms of MRKH.

## Materials and Methods

### Patient Cohort

The selection of patients for this study was done on the basis of the presence of uterine rudiments with endometrium, since not all MRKH patients have uterine rudiments. Endometrial samples from rudimentary uterine tissue from patients with MRKH syndrome and uterine tissue from healthy controls have been collected since 2005 at the Department of Obstetrics and Gynaecology of the University of Tübingen and stored in liquid nitrogen. The uterine rudiments were macroscopically evaluated for the presence of endometrial tissue by a pathologist. If present, the entire endometrial tissue with possible remnants of myometrial tissue (MRKH and control group) got scraped off the underlying myometrium using a scalpel and flash-frozen in liquid nitrogen and was later used in its entirety for RNA isolation. For this study, we collected the endometrial tissue from 39 patients with MRKH syndrome (22 MRKH type 1 and 17 MRKH type 2, see Supplementary Tables S1, S2) at the time of laparoscopically assisted creation of a neovagina (Brucker et al., 2008). Vaginoplasty and recent reconstructive techniques are the main clinical intervention from which tissues for MRKH syndrome studies are usually collected (Nodale et al., 2014b; Benedetti Panici et al., 2015). As control, the endometrium of 30 premenopausal patients, less than 38 years of age, who underwent hysterectomy for benign disease (e.g., uterine leiomyomas or fibroids), was included in the study (Supplementary Tables S1, S2). Correlation with the individual cycle phase was achieved by taking standardized histories and by using hormone profiles from peripheral blood taken 1 day before surgery (see below). The study received prior approval by the Ethics Committee of the Eberhard-Karls-University of Tübingen (Ethical approval AZ 397/2006, Nr.28/2008BO1, 205/2014BO1).

### Hormone Levels and Correlation With Cycle Phase

Whole blood was taken from patients and controls 1 day before or after surgery. Blood serum was used to measure LH, FSH, P, and E2 with a chemiluminescence immunoassay (Vitros eci; Diagnostic Product Cooperation). Cycle phase 1 (proliferative phase) was assigned when *P* < 2.5 ng/ml, cycle phase 2 (secretory phase) when *P* > 5 ng/ml and the LH:FSH ratio was >1.5 according to the standard of our central laboratory.

### RNA Isolation and Sequencing

Total RNA from endometrium of rudimentary uterine tissue or normal uterus was isolated using the RNeasy Mini Kit (Qiagen) and used for paired-end RNA-seq. Quality was assessed with an Agilent 2100 Bioanalyzer. Samples with high RNA integrity number (RIN > 7) were selected for library construction. Using the NEBNext Ultra II Directional RNA Library Prep Kit for Illumina and 100 ng of total RNA for each sequencing library, poly(A) selected paired-end sequencing libraries (101 bp read length) were generated according to the manufacturer’s instructions. All libraries were sequenced on an Illumina NovaSeq 6000 platform at a depth of around 40 mio reads each. Library preparation and sequencing procedures were performed by the same individual, and a design aimed to minimize technical batch effects was chosen.

### Quality Control, Alignment, and Differential Expression Analysis

Read quality of RNA-seq data in FASTQ files was assessed using *FastQC* (v0.11.4) (Andrews, 2010) to identify sequencing cycles with low average quality, adapter contamination, or repetitive sequences from PCR amplification. Reads were aligned using *STAR* (v2.7.0a) (Dobin et al., 2013) allowing gapped alignments to account for splicing against the *Ensembl* H. sapiens genome v95. Alignment quality was analyzed using *samtools* (v1.1) (Li et al., 2009). Normalized read counts for all genes were obtained using *DESeq2* (v1.26.0) (Love et al., 2014). Transcripts covered with less than 50 reads (median of all samples) were excluded from the analysis leaving 15,131 genes for determining differential expression. Surrogate variable analysis (sva, v3.34.0) was used to minimize unwanted variation between samples (Leek et al., 2012). We set | log_2_ fold-change| ≥ 0.5 and BH-adjusted *p*-value ≤ 0.05 to call differentially expressed genes. Gene-level abundances were derived from *DESeq2* as normalized read counts and used for calculating the log_2_-transformed expression changes underlying the expression heatmaps for which ratios were computed against mean expression in control samples. Read counts provided by *DESeq2* also went into calculating nRPKMs (normalized Reads Per Kilobase per Million total reads) as a measure of relative gene expression (Srinivasan et al., 2016).

#### Gene Annotation, Enrichments, and Regulator Analyses

*G:Profiler2* (v0.1.7) was employed to identify overrepresented Gene Ontology terms for differentially expressed genes (Raudvere et al., 2019). Upstream regulators as well as predicted interactions among DEGs were derived from *Ingenuity Pathway Analysis* (IPA, v01–16, Qiagen). *Cytoscape* was used for visualizing networks (Shannon et al., 2003). Transcription factor binding site analyses were carried out in *Pscan* (v1.4) (Zambelli et al., 2009) on the H. sapiens genome considering −450 to +50 bp of promoter regions for motifs against the JASPAR 2018_NR database. *TFEA.chip* (v1.6) was employed with default parameters to determine transcription factor enrichments using the initial database version of ChIP-Seq experiments (Puente-Santamaria et al., 2019). Cell type-specific endometrial marker genes were taken from a preprint (Wang et al., 2019).

### Co-expression Analysis

Weighted Gene Co-expression Network Analysis (Zhang and Horvath, 2005) was used to identify gene co-expression. WGCNA is based on the pairwise correlation between all pairs of genes in the analyzed data set. As correlation method, biweight midcorrelation (Wilcox, 2005) was used with *maxPOutliers* = 0.1, thereby minimizing the influence of potential outliers. Correlations were transformed in a signed hybrid similarity matrix where negative and zero correlations equal zero, while positive correlations remain unchanged. This similarity matrix was raised to the power β = 7 to generate the network adjacency and thereby suppressing low correlations that likely reflect noise in the data. For a measure of interconnectedness, adjacency was transformed into a topological overlap measure (TOM) that is informed by the adjacency of every gene pair plus the connection strength they share with neighboring genes. 1-TOM was then given as an input to hierarchical clustering which identified modules, i.e. groups of co-expressed genes by applying the *Dynamic Tree Cut* algorithm (Langfelder et al., 2008). Each of these modules was summarized by its first principal component referred to as its eigengene, providing a single value for a module’s expression profile. In order to identify modules affected in MRKH, eigengenes were correlated with the disease trait. A joint Bayesian-frequentistic algorithm combining Bayes Factor (BF) (Wetzels and Wagenmakers, 2012) and significance of a correlation was used to identify modules associated with disease status. Modules with an eigengene-trait correlation of *p*_*Bonferroni*_ ≤ 0.05 | BF ≥ 3 were considered significantly associated with MRKH.

#### Reverse Transcription Quantitative PCR (RT-qPCR)

Equal amounts of total RNA (0.5 μg) were reverse transcribed by using the *Thermo Scientific MaximaTM H Minus cDNA Synthesis Master Mix* (Thermo Fisher Scientific) for RT-qPCR and the resulting cDNA used as template (5 ng) in RT-qPCR analysis. Gene expression was analyzed using *PowerUp SYBR Green Mastermix* (Thermo Fisher Scientific, Waltham, MA, United States) on the *QuantStudio 3 Real-Time PCR system* (Thermo Fisher Scientific, Waltham, MA, United States). All experiments were performed in triplicates and relative quantification was done using the 2^−Δ*Ct*^ method. Relative expression was normalized against the house-keeping genes *GAPDH* and *UBE4A*. Primers used for gene expression analysis are listed in Supplementary Table S3.

## Results

### Widespread Transcriptome Changes in Endometrial Tissue of MRKH Patients

To investigate disease-associated perturbations in the endometrial transcriptome of MRKH patients, we performed RNA-seq of uterine rudiments obtained from 39 patients (22 type 1 and 17 type 2) as well as 30 controls. Throughout the analysis pipeline, stringent quality filters were applied, which also helped to identify a few outlier samples using clustering techniques (Supplementary Figures S1A,B). As no obvious sequencing parameters differed for these samples and no batch effects were apparent, tissue composition differences resulting from the sample collection process were considered. After integration of the data with single-cell data from endometrial tissue that recently became available (Wang et al., 2019), the expression signature of cell type-specific markers, in particular for ciliated and unciliated epithelial cells, indeed distinguished the outlier samples from all other samples (Supplementary Figure S1C) and suggested a different underlying cell type composition. Since it is difficult to assess the combinatorial complexity and effects of these convolutions computationally, the affected samples were removed from all subsequent analyses, which left a total of 60 high-quality samples with consistent expression signatures (Supplementary Figure S2).

**FIGURE 1:**
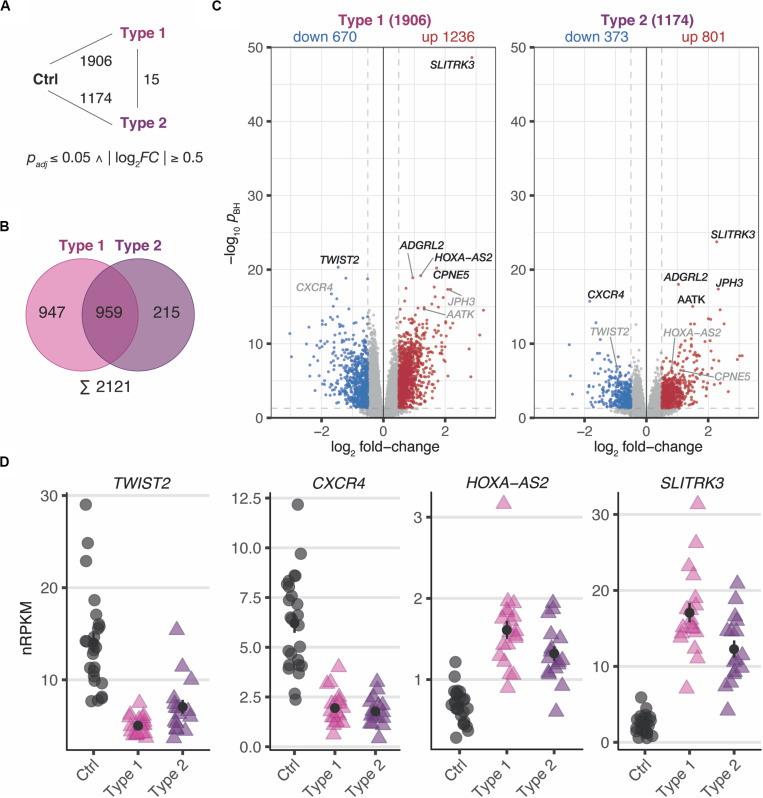
MRKH patients exhibit wide-spread gene expression changes in endometrial tissue compared to unaffected controls. **(A)** Schematic diagram of three experimental groups (Ctrl, unaffected women; Type 1, patients with MRKH type 1; and Type 2, patients with MRKH type 2) indicating number of differentially expressed genes (DEGs) for each pairwise comparison. Fold change and significance cut-offs below. **(B)** Venn diagram comparing common and distinct DEGs between MRKH types 1 and 2. **(C)** Volcano plot showing magnitude and significance values of gene expression changes identified in endometrial tissue of MRKH type 1 **(left panel)** and MRKH type 2 patients **(right panel)** compared to unaffected controls. Blue and red dots highlight DEGs according to the applied cut-offs (see **panel A**). The five most significant DEGs from each comparison are labeled. Gray gene labels indicate location of DEGs from the other comparison. **(D)** Expression levels for the most significant up- and downregulated DEGs as well as the lincRNA *HOXA-A2* plotted as individual data points with mean ± SEM.

In a first step, differential expression changes were determined between MRKH patients and control samples. According to thresholds of *p*_*BH*_ ≤ 0.05 and | log_2_*FC*| ≥ 0.5, a total of 1906 differentially expressed genes (DEGs) comprising 1236 up- and 670 downregulated genes in MRKH type 1 and 1174 DEGs with 801 up- and 373 downregulated genes in MRKH type 2 were identified when compared to controls (Figure 1A). These numbers of affected genes in each disease type indicate profound transcriptome changes in the endometrium of MRKH patients.

### Largely Similar Endometrial Expression Profiles in MRKH Types 1 and 2 Patients

Next, the DEG sets of each pairwise contrast were compared in order to better understand common and distinct expression changes for the disease subtypes. While overlapping the DEGs by name, about half of them first seemed exclusive for type 1 or type 2, respectively (Figure 1B). Directly contrasting the subtypes in the differential analysis, however, identified only 15 DEGs (Figure 1A and Supplementary Figure S3), which suggested largely comparable perturbations in type 1 and 2.

**FIGURE 2:**
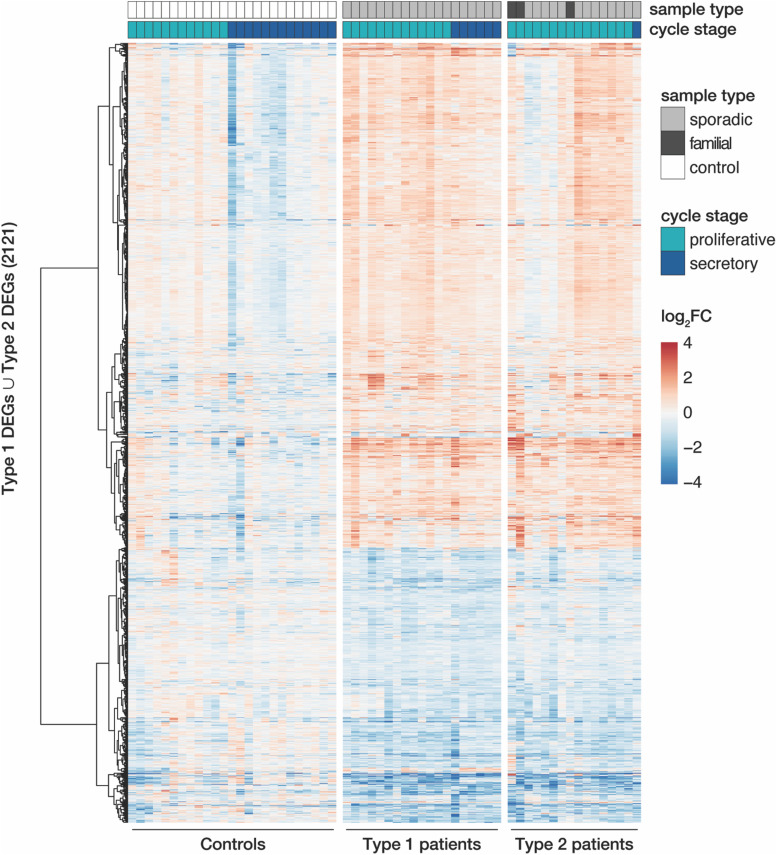
MRKH types 1 and 2 patients show largely similar perturbation patterns in endometrial gene expression. Expression profiles (log_2_ expression change relative to Ctrl group) of 2121 DEGs (union of DEGs indicated in Figure 1B) across all samples. Rows hierarchically clustered by Euclidian distance and *ward.D2* method. Cycle information (proliferative or secretory) and patient type (sporadic, familial, or control) on top. For details see Supplementary Table S1.

Despite similar magnitudes of expression changes in both disease types, affected genes in type 2 samples separated less significantly from controls (Figure 1C and Supplementary Tables S4, S5), pointing to a larger variability among type 2 samples and coinciding with the greater heterogeneity of clinical features in this disease type. Yet, the localization of the top most significant DEGs in the volcano plot (Figure 1C) as well as the correlation of expression changes (spearman rank, *r* = 0.87, see top 50 most significantly up- and downregulated genes in Supplementary Tables S4, S5) pointed at stark similarities between both types. Indeed, the most significant DEGs showed nearly identical expression changes on a gene and transcript isoform level in both disease types (Figure 1D and Supplementary Figure S4A). These changes were further confirmed by RT-qPCR for the most significant DEGs (Supplementary Figure S5). The high degree of concordance also became apparent from the per-sample expression profiles for the union of all DEGs (Figure 2). Further, the expression changes were comparable between sporadic cases and patients from families with more than one affected sibling (Figure 2). Hence, all subsequent analyses were based on the genes underlying this perturbance signature.

#### Endometrial Gene Expression Changes During the Menstrual Cycle Are Disrupted in MRKH Patients

Upon closer inspection of the perturbation signature, the heatmap also showed patterning between the proliferative and secretory cycle stage in control samples for a subgroup of genes (upper part of Figure 2). In MRKH patients, however, this menstrual cycle dependency seemed largely lost. To better quantify this observation, we determined differential expression between the proliferative and secretory phase in control samples, which yielded 818 DEGs (Supplementary Figure S6A). Their associated gene ontology (GO) terms were enriched most significantly for *collagen-containing extracellular matrix* (Supplementary Figure S6B), agreeing with remodeling processes of the extracellular matrix along the transitions between cycle stages (Ruiz-Alonso et al., 2012). In contrast, only 116 genes were identified as cycle-dependent in MRKH type 1 (Supplementary Figure S6A), indicating that cyclic expression adaptations were damped or lost in these patients despite normal hormone profiles (Supplementary Table S1). Instead, the expression of cycle-dependent genes seemingly remained in the proliferative phase throughout the menstrual cycle (Supplementary Figure S6C). The analogous analysis for type 2 was omitted due to the highly skewed sample distribution with respect to cycle stages. Together, these analyses are in line with previous reports that the endometrium of MRKH patients does not respond correctly to cycle hormones (Ludwig, 1998a,b; Rall et al., 2013; Brucker et al., 2017).

### Transcriptome Changes Point to Regulators of Cell Adhesion and Development

**FIGURE 3:**
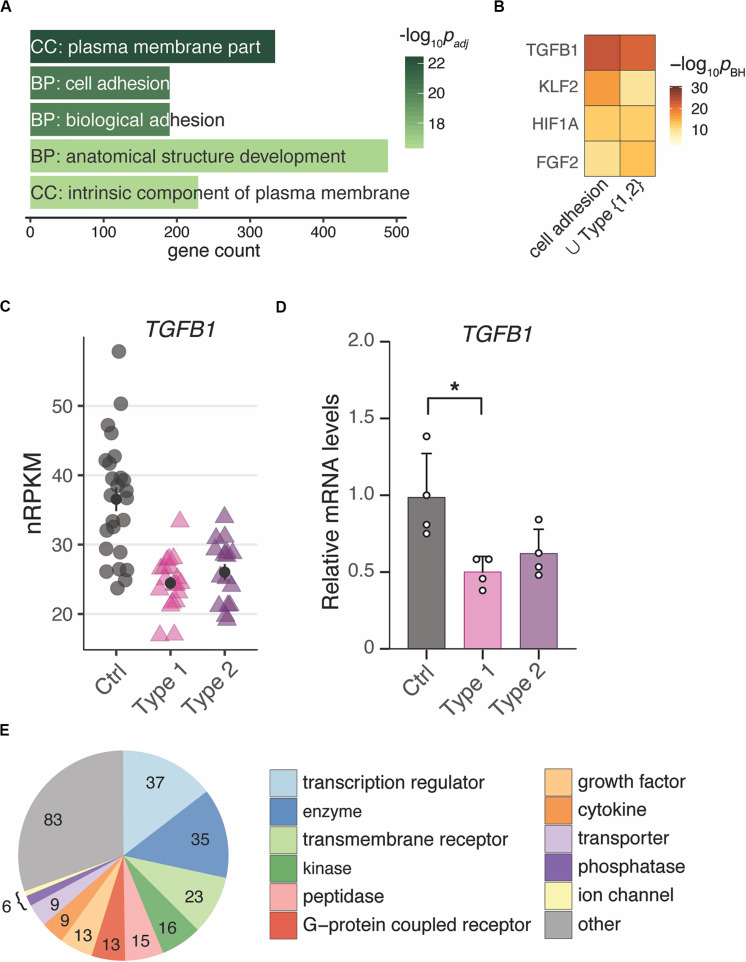
Changes of gene expression in both types of MRKH point to regulators of cell adhesion and development. **(A)** Enrichment analysis identified several significantly overrepresented Gene Ontology terms among the 2121 DEGs (union indicated in Figure 1B). Top five terms with number of associated genes are shown according to their significance. CC, cellular compartment; BP, biological process. **(B)** Comparison of predicted upstream regulators for the DEGs underlying the cell adhesion term (see **panel A**) as well as all 2121 DEGs (union from Figure 1B) based on Ingenuity Pathway Analysis. Top three significant regulators for each gene set shown. **(C)** Expression changes for *TGFB1* plotted as individual data points with mean ± SEM. **(D)** RNA-seq results of *TGFB1* were verified by RT-qPCR. mRNA levels relative to controls plotted as individual data points with mean ± SEM. Significances based on one-way ANOVA with *p < 0.05. **(E)** Among the 253 predicted interactors of TGFB1 differentially expressed in both types of MRKH, transcriptional regulators represent a largest subgroup. Interactors identified based on Ingenuity Pathway Analysis.

To unravel the underlying biology of the endometrial MRKH signature, enrichment analyses were applied to identify potential key regulators as well as affected pathways and cellular processes. With respect to GO terms, *plasma membrane part* was the most overrepresented cellular comportment, and *cell adhesion* and *biological adhesion* emerged as most significant biological processes followed by *anatomical structure development* (Figure 3A).

Based on binding-site analyses, motifs of the differentially expressed transcription factors *EGR1* and *KLF9* were most significantly overrepresented among the DEGs (Supplementary Figures S7A,B). In addition, approaches that integrate ChIP-seq data into such analyses and thereby account also for indirect binding events and factors with less clear motifs (Puente-Santamaria et al., 2019), suggested the DEGs to be highly enriched for *EZH2* targets (Supplementary Figures S7C,D). EZH2 (Enhancer of zeste homolog 2), a histone methyltransferase and a catalytic component of PRC2, showed a trend toward up-regulation in MRKH patients (Supplementary Figure S7E).

To extend the transcription factor-centered analyses to other regulatory mechanisms underlying the observed gene expression changes, we used curated interactome data and mined for regulatory enrichments. From these analyses, *TGFB1* was predicted to be the top upstream regulator for the entire DEG set as well as for the subset of DEGs underlying *cell adhesion* as the most likely affected biological process (Figure 3B). *TGFB1* showed a down-regulation that resulted predominantly from the longer protein-coding transcript isoform and was confirmed by RT-qPCR (Figures 3C,D and Supplementary Figure S4B). Intriguingly, TGFB1 is known to interact not only with EGR1, KLF9, and EZH2, but also connects to more than ten percent of all DEGs (253 of 2121), many with regulatory capacity, too (Figure 3E). These results hint at the regulatory neighborhood of TGFB1 as a key modulator of gene expression changes in MRKH.

### Co-expression Analysis Ranked Disease Relevance of TGFB1 Interactors

To further assess the regulatory relevance of the TGFB1 neighborhood identified along the differential expression analysis, in the next step, a co-expression approach was employed in order to capture groups of genes that change and often function together (Zhang and Horvath, 2005).

**FIGURE 4:**
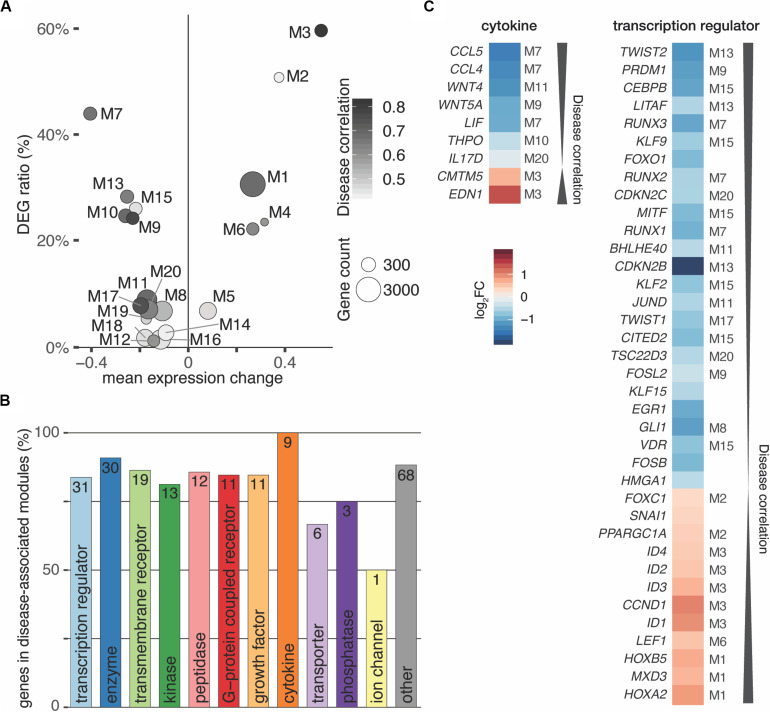
Interactors of TGFB1 reach in all disease-associated co-expression modules. **(A)** Weighted gene correlation network analysis (WGCNA) identified 35 co-expression modules of which 20 were significantly associated with the disease. **(B)** Bar diagram depicting the number of TGFB1 interactors in disease-associated modules. Absolute number within bar as well as amount in percent shown on *y*-axis for each functional type. **(C)** Cytokines and transcriptional regulators predicted to interact with TGFB1 are in highly disease-associated co-expression modules. Module of interactors indicated for significant modules only.

Partitioning of the perturbed expression space using weighted correlation network analysis (Langfelder and Horvath, 2008) led to 35 co-expression modules that ranged from 39 to 3,268 genes in size and totaled to 15,361 genes (Supplementary Figure S8A). In this manner, the co-expression analysis reduced thousands of genes to a relatively small number of coherent modules that represent distinct transcriptional responses. To quantify the overall relationship between modules and the disease, correlations with module eigengenes (summary expression profiles) were calculated (Langfelder and Horvath, 2008). After filtering and correcting with *p*_*Bonferroni*_ ≤ 0.05 and Bayes factor ≥ 3, twenty modules (6 up- and 14 downregulated) passed the significance cut-off (Figure 4A and Supplementary Figure S8B). Furthermore, the meta-analysis significance statistics ranks modules by their overall association with the disease (Figure 4A) and yields a measure of module membership for all genes in all modules. The module membership measures how similar the gene expression profile is to a module’s eigengene. Genes whose profiles are highly similar to the eigengene are considered hub genes and have been shown to implicate relevant biological functions (Langfelder and Horvath, 2008).

According to these characteristics, *TGFB1* located to the disease-associated module M13, correlated significantly with the disease (*r* = 0.68, *p* ≈ 10^–9^), and was among the top 50 hub genes of this module. Of the 253 TGFB1 interactors, 214 reached into all 20 significant disease-associated modules (Figure 4B). Genes annotated for *transcriptional regulator* constituted the largest subgroup of interactors, accompanied by all interacting *cytokines* found in high-ranking modules (Figures 4B,C). Among them were *WNT4* and *WNT5A* of the *WNT signaling* pathway as well as *HOXA2* and *HOXB5* as members of the HOX clusters (Figure 4C and Supplementary Figure S9), all of which have been associated with MRKH (Ledig and Wieacker, 2018). In addition, *TWIST2* identified as one of the genes with the most significant expression change (Figure 1D) ranked highest among the interacting transcription regulators.

### Transcriptome Changes in MRKH Converge on Regulatory Loops in the TGFB1 Neighborhood

The combined approach of differential and co-expression analyses highlighted candidate genes that can explain large parts of the altered expression landscape in context of the MRKH syndrome. These novel candidates together with previously associated genes like *FOXO1* (Demir Eksi et al., 2018) and pathways like *WNT signaling* (Ledig and Wieacker, 2018) share direct links into the TGFB1 regulatory neighborhood (Attisano and Wrana, 2013) and reached into disease-associated co-expression modules.

**FIGURE 5:**
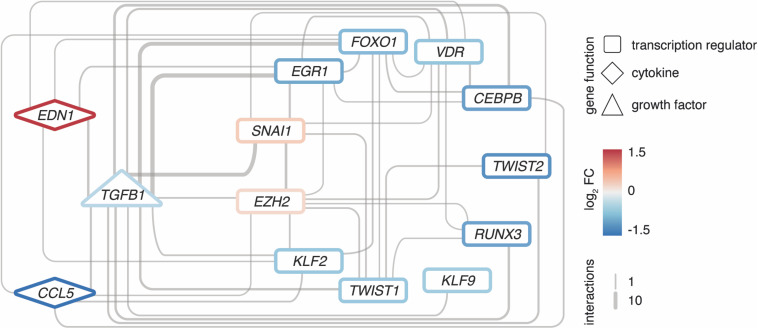
Regulatory loops around TGFB1 link important transcription factors and cytokines. Network of EZH2 as well as upstream interactors of TGFB1 among the 2121 DEGs, respectively. Interactions based on Ingenuity Pathway Analysis and filtered for transcription regulators and cytokines. All interconnections between genes shown. Genes color-coded by mean expression change observed in MRKH/Ctrl. Line width indicates number of curated interactions.

Intriguingly, many interactors are not only targets of TGFB1, but often also upstream regulators, hence, forming regulatory loops (Figure 5). Along such loops, the predicted transcriptional regulators EGR1, KLF9, and EZH2 are found, too. These loops are connected to a dense core network that emerged as pivotal in explaining the disease signature and comprised some of the most significantly altered genes with potent regulator capacity like *TWIST2*.

To further disentangle the regulatory relations in the core network towards a potential point of origin, information from other regulatory layers or functional experiments is required. With respect to the former, epigenomic interrogations might yield additional insight given the prominent location of EZH2 and its role in development. With respect to the latter, the network might serve as starting point to select candidate genes for functional characterizations. To facilitate the selection process and put choices into perspective with respect to other gene expression changes, we implemented an online tool that allows downloading, visualizing, and navigating through the endometrial transcription landscape from single genes to entire pathways: http://mrkh-data.informatik.uni-tuebingen.de.

## Discussion

In this study, we assembled a large cohort of patients with type 1 or type 2 MRKH syndrome and profiled the endometrial transcriptome. The key goal of these efforts was to gain a genome-wide understanding of expression changes in order to identify dysregulations and potential origins of the disease, as only a fraction of MRKH cases can be traced to genetic defects.

Our analyses first revealed widespread perturbations of gene activity in the endometrium that were highly similar between type 1 and type 2 patients. The genes underlying this shared perturbance signature point to key regulators that are centrally linked to cell adhesion and developmental processes. Previous microarray interrogations of myometrial tissue did either not distinguish between the two types in their analysis (Rall et al., 2011) or found a large overlap of gene expression changes between both types with few type-specific expression changes for genes such as *HOXC8* (Nodale et al., 2014a).

Despite highly similar expression perturbations, phenotypically MRKH type 1 and type 2 patients differ. Type 1 cases are characterized by utero-vaginal malformations only, while type 2 patients display a more complex phenotype that entails non-genital abnormalities. Specifically, the urogenital tract including the kidneys is frequently affected in type 2 (e.g. unilateral kidney agenesis, ectopia of one or both kidneys, and horseshoe kidneys). Furthermore, skeletal anomalies, hearing defects, cardiac and digital anomalies as well as ciliopathies occur in type 2 cases (Rall et al., 2015). The utero-vaginal malformations, however, are highly similar between both disease types. Uterus rudiments exist in both, although to a lesser extent in type 2.

As the innermost lining layer of the uterus, the endometrium consists of multiple cell types in a basal and functional tissue layer. As the latter thickens and is shed during menstruation, the endometrium undergoes substantial modifications during the proliferative, secretory, and menstrual phase. The correct staging of these phases is governed by cyclic gene activity over the course of the menstrual cycle (Ruiz-Alonso et al., 2012). In line, we observed expression changes between the proliferative and secretory phase in control samples. Intriguingly, these were largely lost in MRKH patients. Instead, the expression of most genes remained in the proliferative phase although the hormonal profiles indicate patients were in the secretory phase. This finding agrees with previous studies that describe lacking responsiveness of the endometrium to hormones in MRKH patients (Ludwig, 1998a,b; Rall et al., 2013; Brucker et al., 2017). The transcriptome data we provide now offer the opportunity to trace the phenomenon to individual genes and pathways and examine co-occurring effects.

Developmentally, the uterus as well as the upper two thirds of the vagina originate from fusion of the Müllerian ducts. In context of MRKH syndrome, the fusion seems inhibited in gestational week eight and only two uterine rudiments and a vaginal dimple are formed (Rall et al., 2013). They remain in this incomplete embryonic stage and do not undergo normal enlargement at the beginning of adolescence. As the malformation manifests early during embryonic development, associated genes such as *HOX*, *WNT*, and those encoding anti-Müllerian hormone (*AMH*) and its receptor have been proposed to be key for MRKH syndrome (Ravel et al., 2009; Sultan et al., 2009). In particular, *WNT4* is known to be essential for the development of the female reproductive tract and heterozygous mutations in *WNT4* have been associated with a distinct clinical entity of MRKH (Biason-Lauber et al., 2004, 2007; Philibert et al., 2008, 2011). Here, we show that *WNT4* is significantly downregulated in the endometrium of sporadic MRKH patients. Despite very few known mutations so far (Ravel et al., 2009; Liatsikos et al., 2010), alterations in other members of the complex WNT signaling pathway such as *WNT5A*, *WNT7A* and *WNT9B* as well as various *HOX* genes have been suggested as being causative and showed differential expression in our study.

Besides these known key genes for MRKH, we also identified novel candidates that might play a role in MRKH. Among the DEGs, *SLITRK3* stood out to be most significantly upregulated. *SLITRK3* (SLIT And NTRK Like Family Member 3) is expressed predominantly in neural tissues with neurite-modulating activity (Aruga et al., 2003), in hematopoietic stem cells and leukemias (Milde et al., 2007), and correlates to gastrointestinal stromal tumor risk rating and prognosis (Wang et al., 2015). Nevertheless, the human protein atlas also shows strong expression of *SLITRK3* in female tissues, making it a strong candidate to be further followed up in context of MRKH.

On a pathway level, we identified significant enrichments for *cell adhesion* and *anatomical structure development* among perturbed genes. Interestingly, developmental regulators like *TGFB1* and *EZH2* emerged as central from the analyses. *TGFB1* was significantly downregulated in MRKH patients and belongs to the superfamily of transforming growth factor β (TGFβ), which is centrally involved in cell growth and differentiation as well as in regulation of female reproduction and development (Li, 2014). While the uterus of *Tgfb1* mutant mice are morphologically normal, embryos become arrested in the morula stage (Ingman et al., 2006), suggesting critical roles of this gene. Furthermore, TGFβ signaling is crucial for the epithelial to mesenchymal transition (EMT), in which cells lose their epithelial characteristics and acquire migratory behavior (Xu et al., 2009). EMT is necessary for the development and normal functioning of female reproductive organs such as ovaries and uterus and dysregulation may cause endometriosis, adenomyosis, and carcinogenesis (Bilyk et al., 2017).

TGFB1 is linked to Enhancer of zeste homolog 2 (EZH2) (Cardenas et al., 2016; Martin-Mateos et al., 2019; Tsou et al., 2019), the most overrepresented transcriptional regulator predicted to bind to the DEGs according to motif analysis and ChIP-seq reference data. EZH2 is the rate-limiting catalytic subunit of the polycomb repressive complex 2 that silences gene activity epigenetically through deposition of the repressive H3K27me3 histone mark (Laugesen et al., 2016).

In MRKH patients, *EZH2* showed a small but significant trend of upregulation, potentially remains of elevated activity earlier in life. If true, altered levels of EZH2 might have led to falsely deposited H3K27me3 marks in the genome during development which caused perturbations in gene activity and interfered with correct unfolding of the developmental program. The observed transcriptional perturbances at the time of profiling might hence be direct consequences or indirect adaptation attempts of the system. EZH2 has many interaction partners, among them the long non-coding RNA *HOXA-AS2* (HOXA cluster antisense RNA 2) that was significantly upregulated in MRKH patients. Interacting with *HOXA-AS2*, EZH2 represses the transcription of p21 (CDKN1A) and KLF2 thereby influencing cell proliferation (Ding et al., 2017).

In mice, uterine *EZH2* expression is developmentally and hormonally regulated, and loss leads to aberrant uterine epithelial proliferation, uterine hypertrophy, and cystic endometrial hyperplasia (Nanjappa et al., 2019). Furthermore, reduction of EZH2 and ultimately H3K27me3 results in increased expression of estrogen-responsive genes (Bredfeldt et al., 2010).

Intriguingly, prenatal exposures of fetuses to synthetic estrogen such as diethylstilbestrol (DES) can also disturb the development of the female reproductive tract by altering *HOX* gene expression in the developing Müllerian system (Block et al., 2000). During the 1970’s, DES was heavily prescribed to pregnant women to prevent miscarriages and other pregnancy. Much later, it was realized that daughters who were exposed to DES *in utero* showed a higher incidence of Müllerian anomalies (Kaufman et al., 2000) and a higher prevalence of MRKH (Wautier et al., 2019). In this context, exposure to environmental estrogens has also been proposed to reprogram the epigenome by inducing non-genomic ER signaling via the phosphatidylinositol-3-kinase (PI3K) pathway (Walker, 2011). The kinase AKT/PKB phosphorylates and inactivates EZH2 and thereby decreases H3K27me3 levels in the developing uterus. Consequently, estrogen-responsive genes become hypersensitive to estrogen in adulthood and cause hormone-dependent tumors to develop. Our results suggest the opposite effect might play a role in MRKH and failure of enlargement in organ size is a consequence of elevated EZH2 levels.

Taken together and given that only a fraction of MRKH syndrome cases can be explained by genetic defects, these hints toward epigenetic dysregulation playing a potential role in the etiology should be further investigated. Toward these efforts, we consider our results and data to serve as reference point and resource for further exploration.

## Data Availability Statement

The authors acknowledge that the data presented in this study must be deposited and made publicly available in an acceptable repository, prior to publication. Frontiers cannot accept a manuscript that does not adhere to our open data policies.

## Ethics Statement

The studies involving human participants were reviewed and approved by Ethics Committee of the Eberhard Karls University of Tübingen (Ethical approval AZ 397/2006, Nr. 28/2008BO1, 205/2014BO1). The patients/participants provided their written informed consent to participate in this study.

## Author Contributions

SYB, OR, and OK conceived and designed the project. AnK and KR collected and processed the patient samples. TH, NW, and JS-H analyzed the data and developed the online tool. AlK and SB helped with the analyses. AM and AnK performed the RT-qPCR validation. NC was responsible for library preparation and sequencing the samples. TH and JS-H wrote the manuscript. All authors contributed to the interpretation of results and provided critical feedback on preparation of the manuscript.

## Conflict of Interest

The authors declare that the research was conducted in the absence of any commercial or financial relationships that could be construed as a potential conflict of interest.

## Supplementary Material

Supplementary Figure 1 Outlier samples characterized by heterogeneity in cell-type composition. **(A)** Principal component analysis of endometrial gene expression profiles for all initial 69 samples. Axis percentages indicate variance contribution of principle components. **(B)** Correlation map depicting hierarchical clustering of sample-to-sample distances for all initial 69 samples. Variance-stabilized transformed RNA-seq read counts for whole transcriptomes were used to calculate sample-to-sample Euclidean distances (color scale). Results hierarchically clustered using the *complete* method. **(C)** Cell type-specific gene expression per sample for unciliated and ciliated epithelium. Boxplots show geometric mean as well as 10th, 25th, 75th, and 90th quantile of expression values for all genes classified based on single-cell data of the human endometrium (Wang et al., 2019). Number of genes per cell type in brackets.

Supplementary Figure 2 MRKH type 1 and 2 samples separated from control tissue in unaffected individuals. **(A)** Principal component analysis of endometrial gene expression profiles for 60 samples after removal of outliers identified in Supplementary Figure S1. Axis percentages indicate variance contribution. **(B)** Correlation map depicting hierarchical clustering of sample-to-sample distances for the 60 samples remaining after outlier removal. Variance-stabilized transformed RNA-seq read counts for whole transcriptomes were used to calculate sample-to-sample Euclidean distances (color scale). Results hierarchically clustered using the *complete* method. **(C)** Cell type-specific gene expression per sample for unciliated and ciliated epithelium. Boxplots show geometric mean as well as 10th, 25th, 75th, and 90th quantile of expression values for all genes classified based on single-cell data of the human endometrium (Wang et al., 2019). Number of genes per cell type in brackets.

Supplementary Figure 3 Genes differentially expressed between MRKH type 1 and 2. Expression profiles (*log*_2_ expression change relative to Ctrl group) of 15 DEGs differentially expressed between MRKH type 1 and type 2 across all samples. Rows hierarchically clustered by Euclidian distance and *ward.D2* method. Cycle information (proliferative or secretory) and patient type (sporadic, familial, or control) on top. For details see Supplementary Table S1.

Supplementary Figure 4 DEGs showed similar expression changes on transcript isoform level for both types of MRKH. **(A)** Transcript isoform-specific expression changes of *TWIST2*, *CXCR4*, and *SLITRK3* across all conditions. Mean normalized read counts plotted; bold isoforms are protein-coding. **(B)** Transcript isoform-specific expression changes of *TGFB1* across all conditions. Mean normalized read counts plotted; bold isoforms are protein-coding.

Supplementary Figure 5 Validation of gene expression using RT-qPCR. RNA-seq results of most significant DEGs were verified by RT-qPCR. mRNA levels relative to controls plotted as individual data points with mean ± SEM. Significances based on one-way ANOVA with ***p* < 0.01.

Supplementary Figure 6 Gene expression changes during the menstrual cycle are missing in endometrium of MRKH patients. **(A)** Venn diagram comparing menstrual cycle-dependent DEGs between control and MRKH type 1 samples. **(B)** Enrichment analysis identified several significantly overrepresented Gene Ontology terms among the 818 DEGs from the control samples (see **A**). Top five terms with number of associated genes are shown according to their significance. CC: cellular compartment. **(C)** Expression profiles (*log*_2_ expression change relative to Ctrl group) of 818 DEGs differentially expressed between proliferative and secretory controls shown across all samples. Rows hierarchically clustered by Euclidian distance and *ward.D2* method. Cycle information (proliferative or secretory) and patient type (sporadic, familial, or control) on top. For details see Supplementary Table S1.

Supplementary Figure 7 Differentially expressed genes in both types of MRKH are enriched for EGR1, KLF9, and EZH2 binding sites. **(A)** Promoter analysis of 2121 DEGs for overrepresented transcription factor binding sites. Depicted are *z*-scores for different position weight matrices of known transcription factors that are themselves among the DEGs. Higher *z*-scores reflect higher enrichment. Shown are the top five significantly enriched matrices. **(B)** Expression levels in normalized reads per kilobase per million (nRPKMs) for *EGR1* and *KLF9* plotted as individual data points with mean ± SEM. **(C)** Enrichment analysis of transcriptional regulators for the 2121 DEGs identified in MRKH patients based on ChIP-seq and DNase-I data according to *TFEA.ChIP*. EZH2 is predicted to bind significantly more genes of the DEGs than in the rest of the genome. Analysis based on default parameters for binding sites <1 kB upstreams including enhancer elements. Each dot represents a ChIP-seq accession, EZH2-related accessions in purple. **(D)** GSEA-like analysis of transcription regulators for 2121 DEGs based on *TFEA.ChIP* with default parameters and binding sites <1 kB upstream, including enhancer elements. Results indicate EZH2 to be the most significantly enriched regulator in the ranked gene set of DEGs. **(E)** Expression levels in normalized reads per kilobase per million (nRPKMs) for *EZH2* plotted as individual data points with mean ± SEM.

Supplementary Figure 8 Co-expression analysis points to disease-associated modules. **(A)** Representation of the hierarchical gene clustering tree leading to 35 modules of co-expressed genes. Leaves correspond to 15,361 genes included in the analysis, and height reflects closeness of individual genes. Lower panel shows colors assigned to each module by the *Dynamic Tree Cut* and modules assigned consecutive to the *Merged Dynamic* method using a dissimilarity threshold of 0.1. **(B)** Disease correlations with the corresponding *p*-values for each module. Each cell contains the correlation coefficient between the expression profile of a module and the disease and is color-coded accordingly. Numbers in brackets indicate associated *p*-values.

Supplementary Figure 9 Gene expression changes for altered candidates of the *WNT signaling* pathway and HOX cluster. Expression levels in normalized reads per kilobase per million (nRPKMs) for differentially expressed genes of the *WNT signaling* pathway and HOX clusters previously linked to MRKH and Müllerian duct development (Ledig and Wieacker, 2018). Plotted as individual data points with mean ± SEM.

Supplementary Table 1 Demographic and clinical characteristics of MRKH patients and controls. The labels are used as following. Δ patient samples excluded from analysis due to deviating tissue composition; ^∗^ two sisters from family A; ° two sisters from family B; N/A, not applicable (hysterectomy control); B = bilateral, L = left, R = right; cycle phase 1 ≜ proliferative phase, cycle phase 2 ≜ secretory phase.

Supplementary Table 2 Summary of demographic and clinical characteristics of MRKH patients and controls used for the analysis. Samples excluded from the analysis due to tissue composition (see Supplementary Table S1) were not included in the calculation of the depicted values.

Supplementary Table 3 List of primer sequences used for RT-qPCR.

Supplementary Table 4 Top 50 up- and downregulated genes in Type 1/Ctrl, ordered by *p*_*adj*_ value.

Supplementary Table 5 Top 50 up- and downregulated genes in Type 2/Ctrl, ordered by *p*_*adj*_ value.
