# Supplementary material for: The Endometrial Transcription Landscape of MRKH Syndrome
Source: Front Cell Dev Biol. 2020 Sep 24;8:572281. doi: 10.3389/fcell.2020.572281 (PMC7542331; doi:10.3389/fcell.2020.572281)
Supplement: Supplementary Figure 1 — Outlier samples characterized by heterogeneity in cell-type composition. (A) Principal component analysis of endometrial gene expression profiles for all initial 69 samples. Axis percentages indicate variance contribution of principle components. (B) Correlation map depicting hierarchical clustering of sample-to-sample distances for all initial 69 samples. Variance-stabilized transformed RNA-seq read counts for whole transcriptomes were used to calculate sample-to-sample Euclidean distances (color scale). Results hierarchically clustered using the complete method. (C) Cell type-specific gene expression per sample for unciliated and ciliated epithelium. Boxplots show geometric mean as well as 10th, 25th, 75th, and 90th quantile of expression values for all genes classified based on single-cell data of the human endometrium (Wang et al., 2019). Number of genes per cell type in brackets. [file Data_Sheet_1.PDF]

Supplementary Figure 1

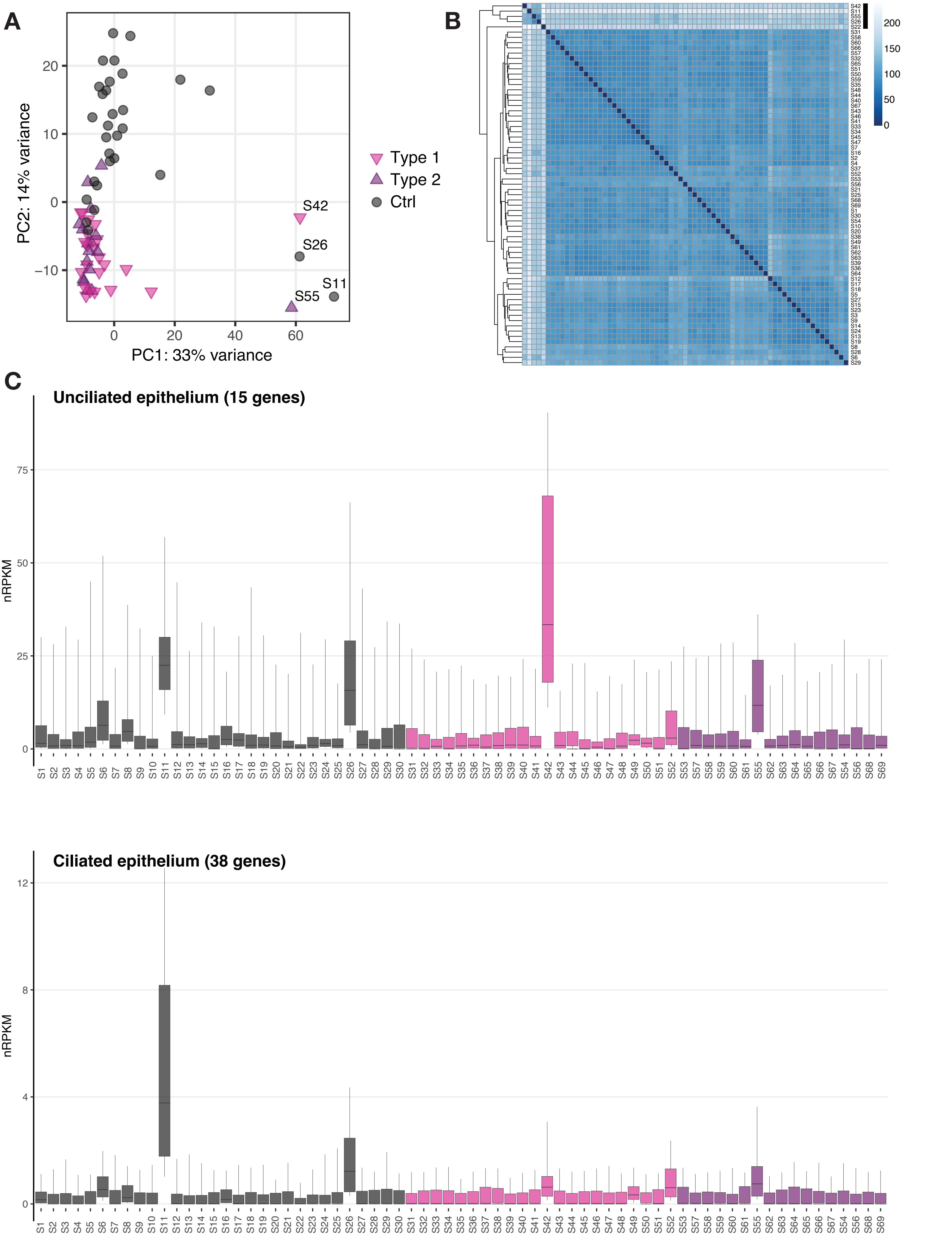

Supplementary Figure 1. Outlier samples characterized by heterogeneity in cell-type composition.

A) Principal component analysis of endometrial gene expression profiles for all initial 69 samples. Axis percentages indicate variance contribution of principle components.

B) Correlation map depicting hierarchical clustering of sample-to-sample distances for all initial 69 samples. Variance-stabilized transformed RNA-seq read counts for whole transcriptomes were used to calculate sample-to-sample Euclidean distances (color scale). Results hierarchically clustered using the *complete* method.

C) Cell type-specific gene expression per sample for unciliated and ciliated epithelium. Boxplots show geometric mean as well as 10th, 25th, 75th, and 90th quantile of expression values for all genes classified based on single-cell data of the human endometrium (Wang et al., 2019). Number of genes per cell type in brackets.

Supplementary Figure 2

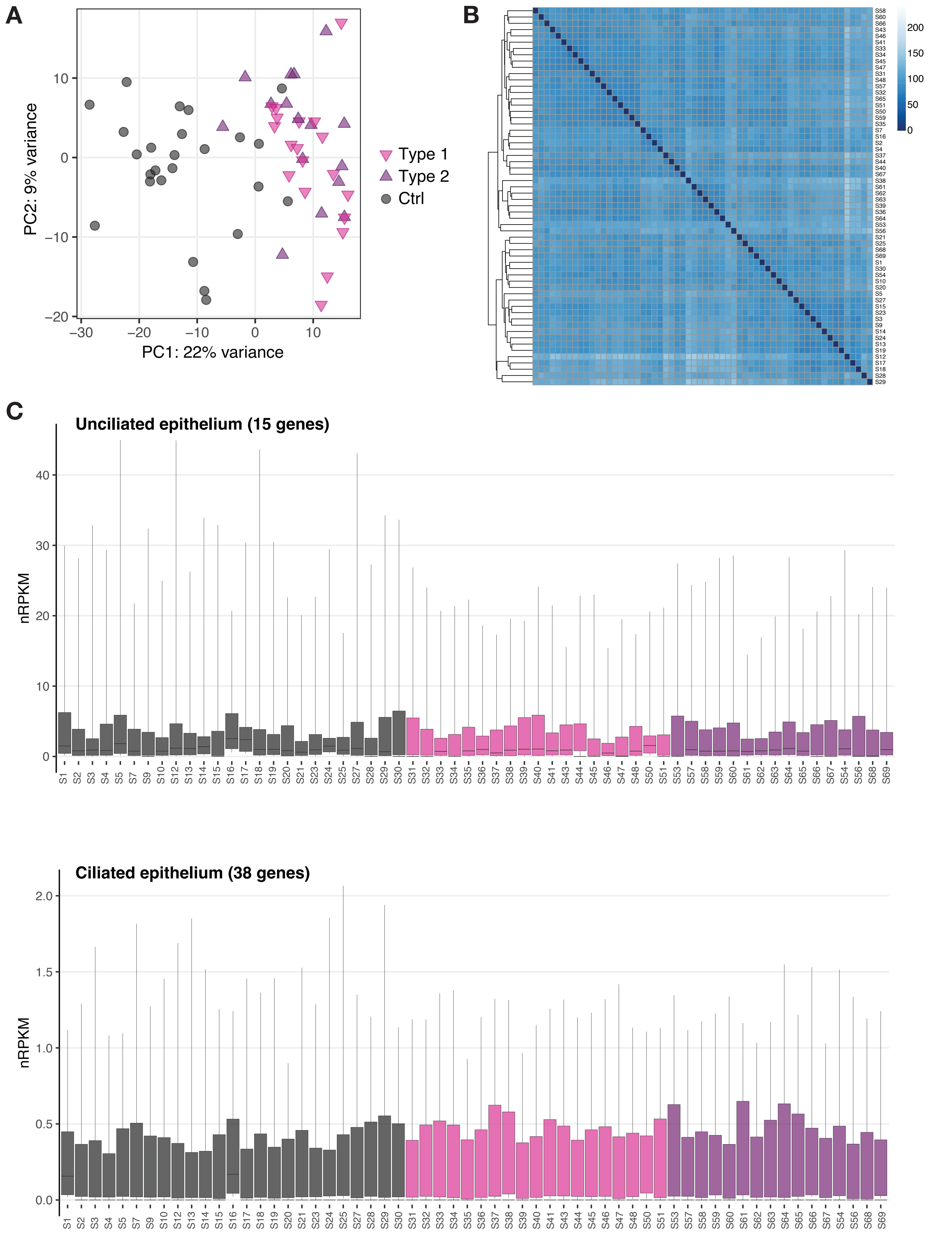

Supplementary Figure 2. MRKH type 1 and 2 samples separated from control tissue in unaffected individuals.

A) Principal cincipal component analysis of endometrial gene expression profiles for 60 samples after removal of outliers identified in Supplementary Figure 1. Axis percentages indicate variance contribution.

B) Correlation map depicting hierarchical clustering of sample-to-sample distances for the 60 samples remaining after outlier removal. Variance-stabilized transformed RNA-seq read counts for whole transcriptomes were used to calculate sample-to-sample Euclidean distances (color scale). Results hierarchically clustered using the complete method.

C) Cell type-specific gene expression per sample for unciliated and ciliated epithelium. Boxplots show geometric mean as well as 10th, 25th, 75th, and 90th quantile of expression values for all genes classified based on single-cell data of the human endometrium (Wang et al., 2019). Number of genes per cell type in brackets.

Supplementary Figure 3

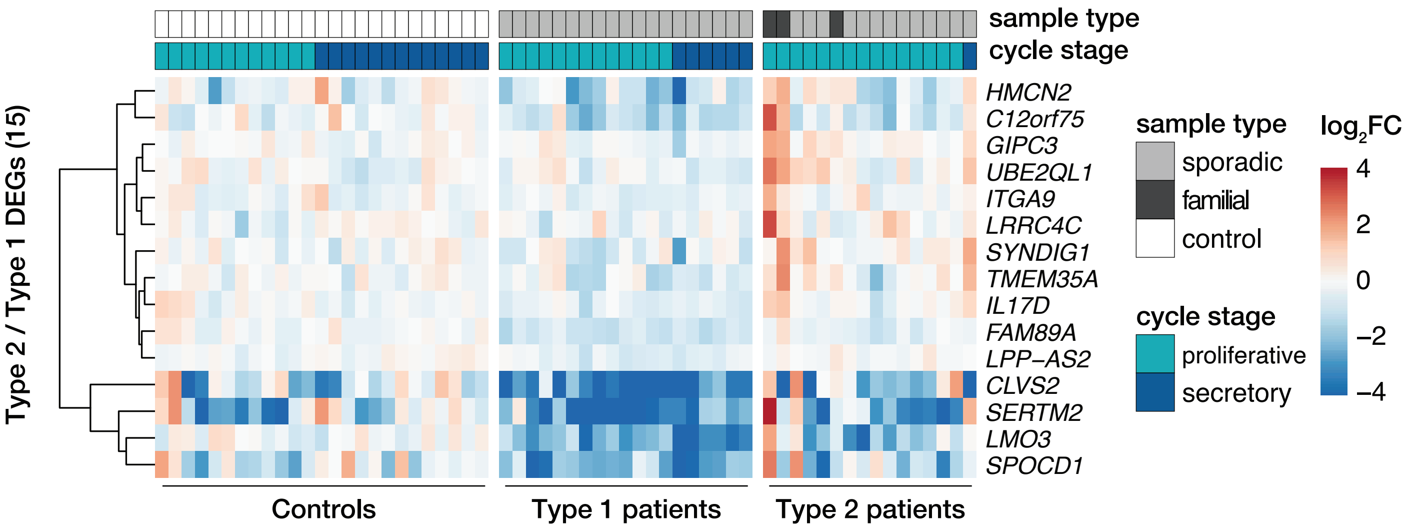

Supplementary Figure 3. Genes differentially expressed between MRKH type 1 and 2.

Expression profiles (*log_2* expression change relative to Ctrl group) of 15 DEGs differentially expressed between MRKH type 1 and type 2 across all samples. Rows hierarchically clustered by Euclidian distance and *ward.D2* method. Cycle information (proliferative or secretory) and patient type (sporadic, familial, or control) on top. For details see Supplementary Table 1.

Supplementary Figure 4

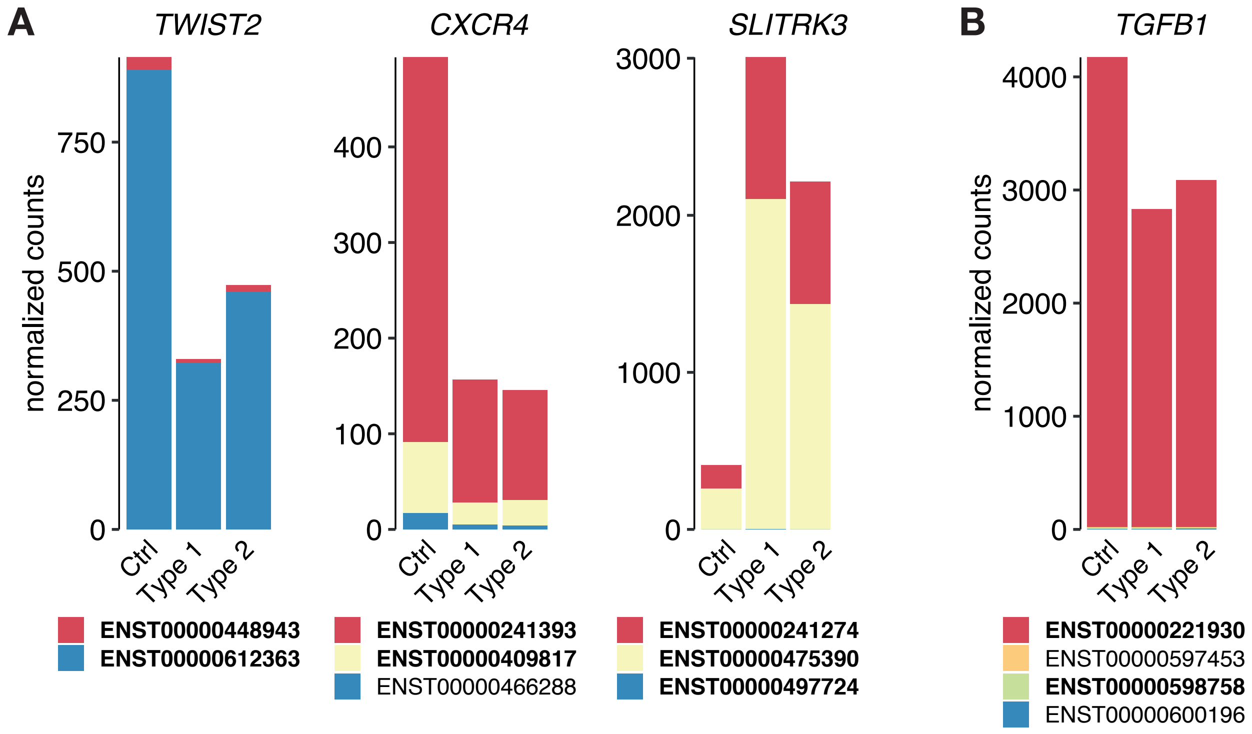

Supplementary Figure 4. DEGs showed similar expression changes on transcript isoform level for both types of MRKH.

- **A)** Transcript isoform-specific expression changes of *TWIST2*, *CXCR4*, and *SLITRK3* across all conditions. Mean normalized read counts plotted; bold isoforms are protein-coding.
- **B)** Transcript isoform-specific expression changes of *TGFB1* across all conditions. Mean normalized read counts plotted; bold isoforms are protein-coding.

Supplementary Figure 5

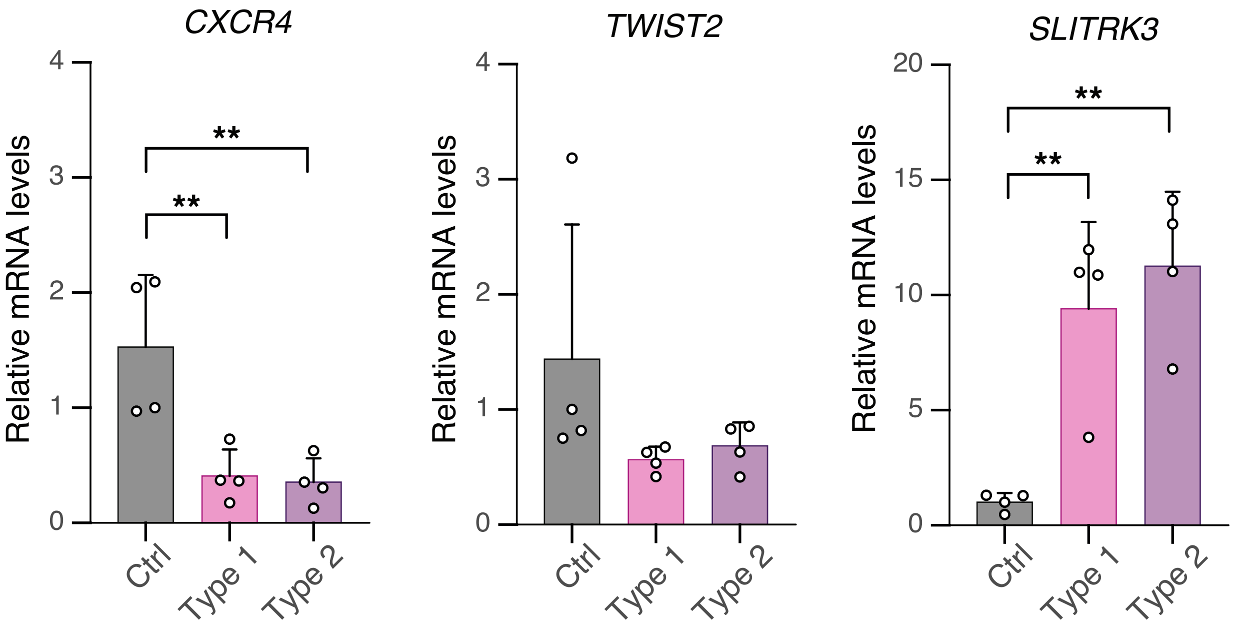

Supplementary Figure 5. Validation of gene expression using RT-qPCR.

RNA-seq results of most significant DEGs were verified by RT-qPCR. mRNA levels relative to controls plotted as individual data points with mean ± SEM. Significances based on one-way ANOVA with $^{**}p < 0.01$.

Supplementary Figure 6

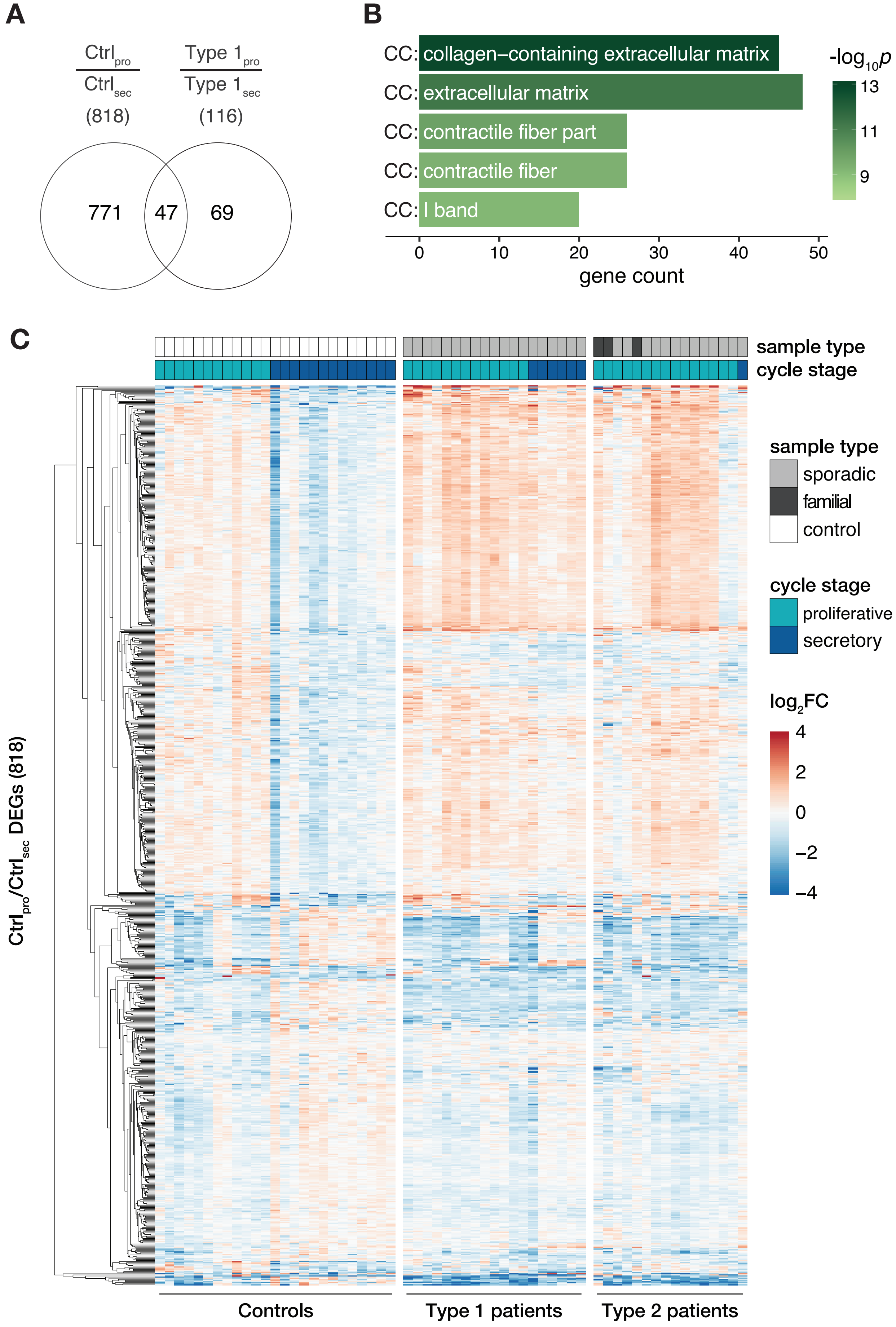

Supplementary Figure 6. Gene expression changes during the menstrual cycle are missing in endometrium of MRKH patients

A) Venn diagram comparing menstrual cycle-dependent DEGs between control and MRKH type 1 samples.

B) Enrichment analysis identified several significantly overrepresented Gene Ontology terms among the 818 DEGs from the control samples (see a). Top five terms with number of associated genes are shown according to their significance. CC: cellular compartment.

C) Expression profiles (log_2 expression change relative to Ctrl group) of 818 DEGs differentially expressed between proliferative and secretory controls shown across all samples. Rows hierarchically clustered by Euclidian distance and *ward.D2* method. Cycle information (proliferative or secretory) and patient type (sporadic, familial, or control) on top. For details see Supplementary Table 1.

Supplementary Figure 7

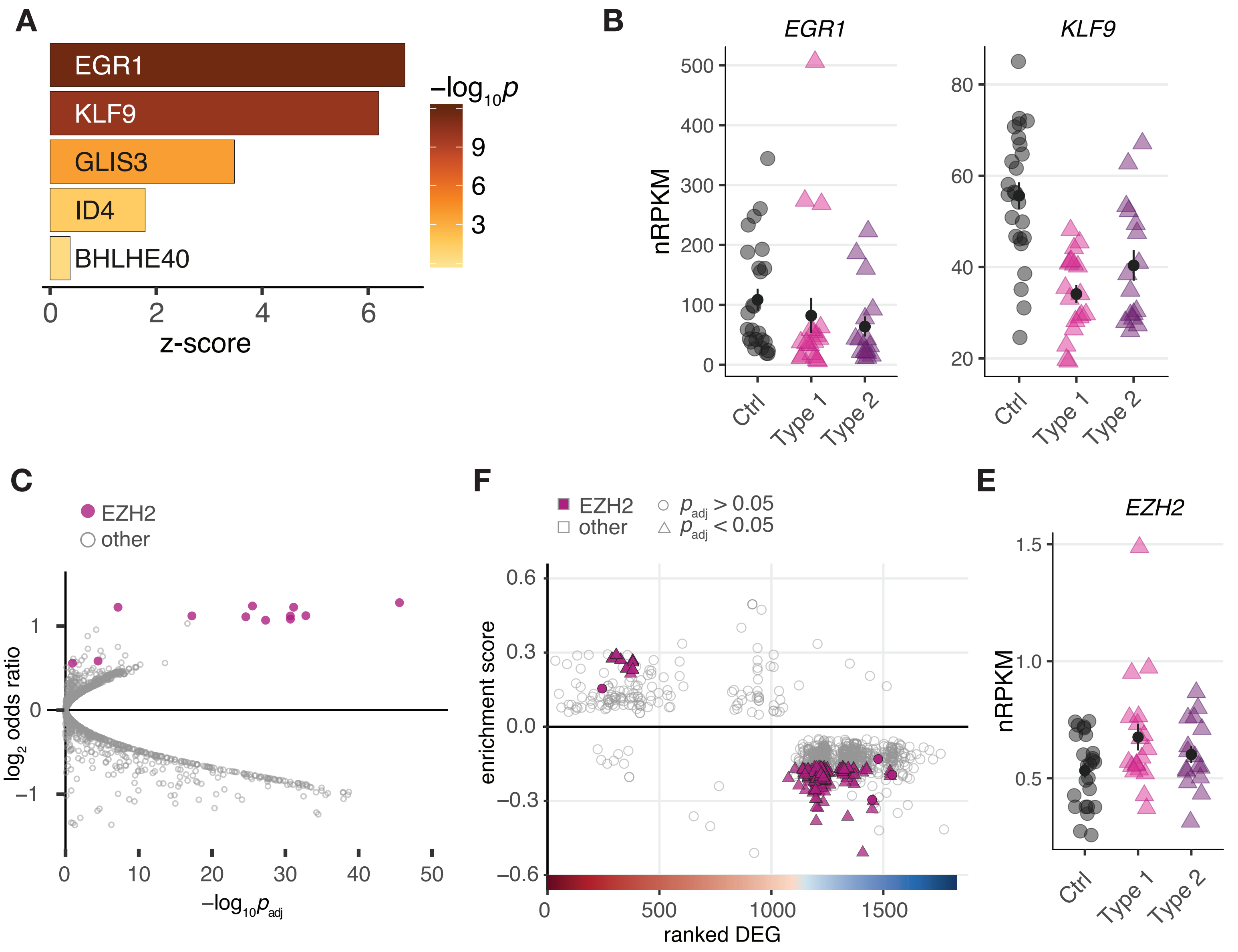

Supplementary Figure 7. Differentially expressed genes in both types of MRKH are enriched for EGR1, KLF9, and EZH2 binding sites.

A) Promoter analysis of 2121 DEGSs for overrepresented transcription factor binding sites. Depicted are z-scores for different position weight matrices of known transcription factors that are themselves among the DEG set. Higher z-score reflect higher enrichment. Shown are the top five significantly enriched matrices.

B) Expression levels in normalized reads per kilobase per million (nRPKMs) for *EGR1* and *KLF9* plotted as individual data points with mean ± SEM.

C) Enrichment analysis of transcriptional regulators for the 2121 DEGs identified in MRKH patients based on ChIP-seq and DNase-I data according to *TFEA.ChIP*. EZH2 is predicted to bind significantly more genes of the DEG set than in the rest of the genome. Analysis based on default parameters for binding sites <1kB upstream including enhancer elements. Each dot represents a ChIP-seq accession, EZH2-related accessions in purple.

D) GSEA-like analysis of transcription regulators for 2121 DEGs based on TFEA.ChIP with default parameters and binding sites <1kB upstream including enhancer elements. Results indicate EZH2 to be the most significantly enriched regulator in the ranked gene set of DEGs.

E) Expression levels in normalized reads per kilobase per million (nRPKMs) for EZH2 plotted as individual data points with mean ± SEM.

Supplementary Figure 8

A

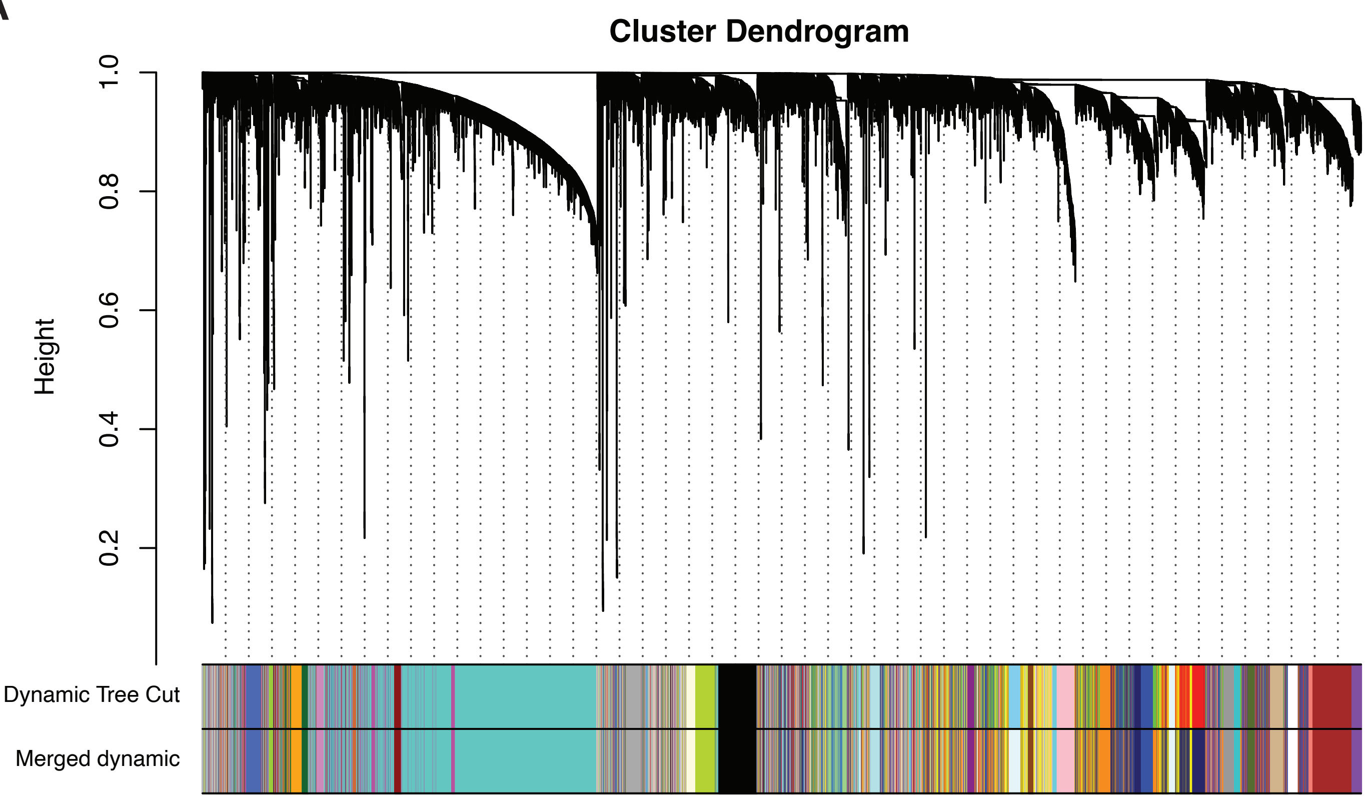

B

Module color	Disease correlation	(p-value)	Module
darkred	0.82	(7e−16)	M3
turquoise	0.69	(2e−09)	M1
orange	0.63	(9e−08)	M6
yellowgreen	0.59	(6e−07)	M4
magenta	0.46	(2e−04)	M5
violet	0.43	(6e−04)	M2
paleturquoise	0.20	(0.1)	
sienna3	0.13	(0.3)	
black	0.13	(0.3)	
royalblue	0.11	(0.4)	
darkgreen	0.08	(0.6)	
grey	−0.06	(0.7)	
darkgrey	−0.09	(0.5)	
cyan	−0.17	(0.2)	
steelblue	−0.19	(0.1)	
darkmagenta	−0.20	(0.1)	
greenyellow	−0.28	(0.03)	
skyblue3	−0.29	(0.03)	
darkolivegreen	−0.32	(0.01)	
brown	−0.33	(0.01)	
tan	−0.35	(0.005)	
purple	−0.43	(7e−04)	M14
midnightblue	−0.45	(3e−04)	M16
lightyellow	−0.45	(3e−04)	M15
pink	−0.46	(2e−04)	M18
saddlebrown	−0.48	(9e−05)	M19
blue	−0.53	(1e−05)	M8
white	−0.6	(5e−07)	M12
yellow	−0.64	(4e−08)	M20
grey60	−0.64	(3e−08)	M13
lightgreen	−0.67	(6e−09)	M7
darkorange	−0.69	(1e−09)	M11
salmon	−0.69	(1e−09)	M10
lightcyan	−0.75	(6e−12)	M17
darkturquoise	−0.77	(5e−13)	M9

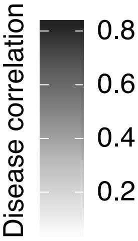

Supplementary Figure 8: Co-expression analysis points to disease-associated modules.

A) Representation of the hierarchical gene clustering tree leading to 35 modules of co-expressed genes. Leaves correspond to 15,361 genes included in the analysis, and height reflects closeness of individual genes. Lower panel shows colors assigned to each module by the *Dynamic Tree Cut* and modules assigned consecutive to the *Merged Dynamic* method using a dissimilarity threshold of 0.1.

B) Disease correlations with the corresponding p-values for each module. Each cell contains the correlation coefficient between the expression profile of a module and the disease (MRKH) and is color-coded accordingly. Number in brackets indicates associated *p*-value.

Supplementary Figure 9

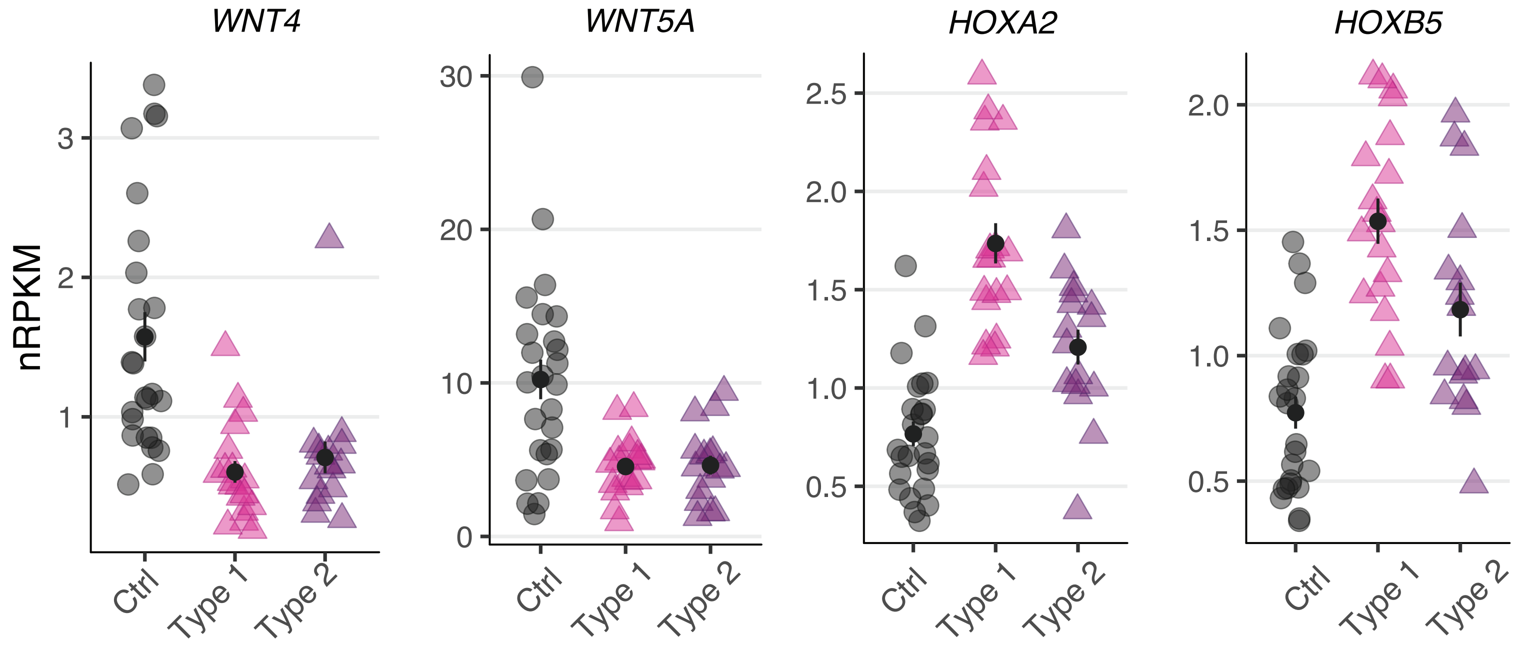

Supplementary Figure 9. Gene expression changes for altered candidates of the WNT signaling pathway and HOX cluster.

Expression levels in normalized reads per kilobase per million (nRPKMs) for differentially expressed genes of the WNT signaling pathway and HOX clusters previously linked to MRKH and the development of the Müllerian duct (Ledig and Wieacker, 2018). Plotted as individual data points with mean ± SEM.

Supplementary Table 1. Demographic and clinical characteristics of MRKH patients and controls

	MRKH type	MURCS	Co-occuring anomalies	Cycle phase
S1	N/A	N/A	N/A	2
S2	N/A	N/A	N/A	1
S3	N/A	N/A	N/A	2
S4	N/A	N/A	N/A	1
S5	N/A	N/A	N/A	1
S6$^{\Delta}$	N/A	N/A	N/A	2
S7	N/A	N/A	N/A	1
S8$^{\Delta}$	N/A	N/A	N/A	2
S9	N/A	N/A	N/A	2
S10	N/A	N/A	N/A	1
S11$^{\Delta}$	N/A	N/A	N/A	2
S12	N/A	N/A	N/A	2
S13	N/A	N/A	N/A	2
S14	N/A	N/A	N/A	2
S15	N/A	N/A	N/A	1
S16	N/A	N/A	N/A	1
S17	N/A	N/A	N/A	2
S18	N/A	N/A	N/A	2
S19	N/A	N/A	N/A	2
S20	N/A	N/A	N/A	1
S21	N/A	N/A	N/A	1
S22$^{\Delta}$	N/A	N/A	N/A	2
S23	N/A	N/A	N/A	1
S24	N/A	N/A	N/A	2
S25	N/A	N/A	N/A	2
S26$^{\Delta}$	N/A	N/A	N/A	1
S27	N/A	N/A	N/A	2
S28	N/A	N/A	N/A	1
S29	N/A	N/A	N/A	2
S30	N/A	N/A	N/A	1
S31	1	No	None	1
S32	1	No	None	1
S33	1	No	None	2
S34	1	No	None	2
S35	1	No	None	1
S36	1	No	None	1
S37	1	No	None	1
S38	1	No	None	2
S39	1	No	None	1
S40	1	No	None	1
S41	1	No	None	2
S42$^{\Delta}$	1	No	None	1
S43	1	No	None	1
S44	1	No	None	1
S45	1	No	None	2
S46	1	No	None	1

S47	1	No	None	2
S48	1	No	None	1
S49$^{\Delta}$	1	No	None	Unknown
S50	1	No	None	1
S51	1	No	None	1
S52$^{\Delta}$	1	No	None	1
S53*	2	Yes	Duplex kidney (R), scoliosis	1
S54*	2	Yes	Duplex kidney (B), complex sceletal malformations	1
S55°$^{\Delta}$	2	No	Scoliosis	1
S56°	2	No	Scoliosis	1
S57	2	No	Duplex kidney (B)	1
S58	2	No	Scoliosis	1
S59	2	No	Ear deformities, auditory canal hypoplasia	1
S60	2	No	Scoliosis	1
S61	2	No	Renal agenesis (R)	1
S62	2	No	Scoliosis	1
S63	2	No	Scoliosis	1
S64	2	Yes	Renal agenesis (L), scoliosis, ear deformities, fistula, imperforate anus	1
S65	2	No	Renal agenesis (R)	1
S66	2	No	Hip dysplasia	2
S67	2	No	Silver-Russell syndrome	1
S68	2	No	Renal agenesis (R)	1
S69	2	No	Nephroptosis (L)	1

$^{\Delta}$ patient samples excluded from analysis due to deviating tissue composition; * two sisters from family A; ° two sisters from family B; N/A , not applicable (hysterectomy control); B = bilateral, L = left, R= right; cycle phase 1 ≙ proliferative phase, cycle phase 2 ≙ secretory phase

Supplementary Table 2. Summary of demographic and clinical characteristics of MRKH patients and controls used for the analysis. Samples excluded from the analysis due to tissue composition (see Supplementary Table 1) were not included in the calculation of the depicted values.

	No of patients	Age at surgery (years), mean ± Stdev	% MURCS	% Renal malformations	% Sceletal malformations	% Further malformations	Cycle phase	
							% phase 1	% phase 2
Controls	25	35.8 ± 1.72	N/A	N/A	N/A	N/A	48,00	52,00
MRKH	35	19.26 ± 5.5	8,57	22,86	28,57	8,57	80,00	20,00
MRKH type 1	19	17.58 ± 2.58	0,00	0,00	0,00	0,00	68,42	31,58
MRKH type 2	16	21.25 ± 7.14	18,75	50,00	62,50	18,75	93,75	6,25

N/A, not applicable (hysterectomy control); cycle phase 1 ≙ proliferative phase, cycle phase 2 ≙ secretory phase

Supplementary Table 3. List of primer sequences used for RT-qPCR

ID	forward	reverse	acession number
TGFB1	TACCTGAACCCGTGTTGCTCTC	GTTGCTGAGGTATCGCCAGGAA	NM_000660
CXCR4	CTCCTCTTTGTCATCACGCTTCC	GGATGAGGACACTGCTGTAGAG	NM_001008540,
TWIST2	GCAAGATCCAGACGCTCAAGCT	ACACGGAGAAGGCGTAGCTGAG	NM_001271893.4
SLITRK3	ACAGACGCCTTTGCTGGCACAT	TCACAGGTGCAGTCCCAAGGAT	NM_001318810
UBE4A	CCTTTGCCAGATGGCTACAGCT	CTCCAGGGAATCTGCTGATGTC	NM_001204077
GAPDH	GACAGTCAGCCGCATCTTCT	TTAAAGCAGCCCTGGTGAC	NG_007073.2

Supplementary Table 4. Top 50 up- and downregulated genes in Type1 / Ctrl, ordered by *padj* value.

ensembl	hgnc_symbol	log2FoldChange_type1	pvalue_type1	padj_type1	log2FoldChange_type2	pvalue_type2	padj_type2
ENSG00000121871	SLITRK3	2,863280258	1,58232E-53	2,39421E-49	2,275486085	1,18182E-28	1,78821E-24
ENSG00000124772	CPNE5	1,721557417	1,20097E-24	6,05731E-21	1,105846163	2,39747E-09	3,85916E-07
ENSG00000253552	HOXA-AS2	1,20846179	1,74413E-23	6,59761E-20	0,816966271	9,38732E-10	1,84467E-07
ENSG00000117114	ADGRL2	0,952064679	4,18797E-23	1,26736E-19	1,036819058	1,30184E-22	9,84908E-19
ENSG00000151892	GFRA1	1,643902733	1,20011E-21	2,26985E-18	1,450410084	1,98284E-14	2,14302E-11
ENSG00000204514	ZNF814	0,731592062	1,97571E-21	3,3216E-18	0,515211446	1,28281E-09	2,33858E-07
ENSG00000185985	SLITRK2	2,172972742	3,3434E-21	4,68135E-18	1,719330986	1,20142E-11	4,54466E-09
ENSG00000154118	JPH3	2,082907384	3,40327E-21	4,68135E-18	2,327879847	8,31723E-22	4,19493E-18
ENSG00000186766	FOXI2	1,675032858	3,96349E-20	4,6132E-17	1,455368069	3,37187E-13	2,31908E-10
ENSG00000213397	HAUS7	1,936793497	5,40138E-20	5,83773E-17	1,597101075	7,28707E-12	3,1503E-09
ENSG00000082512	TRAF5	0,691942627	1,84727E-19	1,74694E-16	0,460476165	5,22799E-08	4,73681E-06
ENSG00000167549	CORO6	1,567207356	4,91984E-19	4,37895E-16	1,014824202	1,65687E-07	1,14476E-05
ENSG00000114200	BCHE	1,779458547	1,46733E-18	1,16853E-15	1,53033441	6,90744E-12	3,07401E-09
ENSG00000092758	COL9A3	1,319956721	1,75118E-18	1,32485E-15	1,252118273	4,11425E-14	3,89079E-11
ENSG00000019102	VSIG2	1,119580194	2,11925E-18	1,52697E-15	0,834836088	3,28135E-09	5,01516E-07
ENSG00000181409	AATK	1,321341694	3,29005E-18	2,16442E-15	1,497059407	3,07538E-19	9,30671E-16
ENSG00000154065	ANKRD29	0,541507288	3,47744E-18	2,19238E-15	0,477558541	3,47853E-12	1,69786E-09
ENSG00000162873	KLHDC8A	3,239385791	4,93346E-18	2,98593E-15	1,941301817	2,55055E-06	8,38964E-05
ENSG00000009724	MASP2	1,032988384	2,47911E-17	1,33969E-14	0,799484811	2,69615E-09	4,24953E-07
ENSG00000100836	PABPN1	0,680400795	9,01018E-17	3,68468E-14	0,378420094	2,729E-05	0,000486366
ENSG00000139190	VAMP1	0,911041004	1,09019E-16	4,34095E-14	0,582194032	1,53506E-06	5,89518E-05
ENSG00000177685	CRACR2B	0,828406365	1,35354E-16	5,25139E-14	0,566945416	2,83544E-07	1,66795E-05
ENSG00000121310	ECHDC2	0,944174081	1,66283E-16	5,99055E-14	0,667162673	1,26068E-07	9,1708E-06
ENSG00000007516	BAIAP3	1,406298549	1,88583E-16	6,63591E-14	0,756388507	6,20258E-05	0,000898098
ENSG00000168993	CPLX1	0,999449222	3,17348E-16	1,05498E-13	0,733846387	5,35677E-08	4,81745E-06
ENSG00000233608	TWIST2	-1,4620495	6,32949E-25	4,78858E-21	-0,941558598	1,58322E-09	2,81831E-07
ENSG00000172216	CEBPB	-1,212403401	5,6826E-23	1,43306E-19	-0,911656986	1,65414E-11	5,82066E-09
ENSG00000122042	UBL3	-0,51032051	8,36932E-23	1,80909E-19	-0,355610539	5,0675E-10	1,09538E-07
ENSG00000121966	CXCR4	-1,683375246	1,52445E-20	1,92221E-17	-1,832988925	4,95067E-20	1,87271E-16
ENSG00000218336	TENM3	-1,506435434	8,54373E-20	8,61835E-17	-1,242473195	9,46506E-12	3,8707E-09
ENSG00000115648	MLPH	-1,621822441	1,06836E-18	8,98073E-16	-1,21486683	1,8832E-09	3,21906E-07
ENSG00000116717	GADD45A	-1,117487482	2,98482E-18	2,05288E-15	-0,702705146	6,53644E-07	3,14977E-05
ENSG00000214717	ZBED1	-0,453595792	5,23469E-18	3,04639E-15	-0,344894651	2,36806E-09	3,85281E-07
ENSG00000109220	CHIC2	-0,487311537	1,19834E-17	6,71559E-15	-0,41976378	2,18263E-11	7,50575E-09
ENSG00000136738	STAM	-0,315698646	2,6447E-17	1,37989E-14	-0,337224827	2,28312E-16	2,65738E-13
ENSG00000185950	IRS2	-1,482077545	4,38662E-17	2,21246E-14	-0,992401118	3,29139E-07	1,87932E-05
ENSG00000057252	SOAT1	-0,512271528	4,73205E-17	2,3097E-14	-0,352029298	1,63719E-07	1,14158E-05
ENSG00000113758	DBN1	-0,484309747	5,13005E-17	2,42571E-14	-0,34358482	6,8545E-08	5,82671E-06
ENSG00000197614	MFAP5	-1,712538831	5,86426E-17	2,68885E-14	-1,33022052	3,72462E-09	5,63572E-07
ENSG00000156504	FAM122B	0,457782107	6,231E-17	2,77298E-14	0,319960441	1,15707E-07	8,58215E-06
ENSG00000172667	ZMAT3	-0,658947703	8,0451E-17	3,47801E-14	-0,545706534	3,89081E-10	8,78685E-08
ENSG00000167378	IRGQ	0,414499309	8,87278E-17	3,68468E-14	0,360958571	5,06722E-11	1,50338E-08
ENSG00000204335	SP5	-1,642372928	1,46689E-16	5,42183E-14	-1,51309355	4,3121E-12	1,97717E-09
ENSG00000136888	ATP6V1G1	-0,454434084	1,46914E-16	5,42183E-14	-0,348341618	9,12144E-09	1,16963E-06
ENSG00000258754	LINC01579	-1,975353374	2,30219E-16	7,91692E-14	-1,747069332	4,60249E-11	1,3928E-08
ENSG00000082781	ITGB5	-0,886641017	4,50074E-16	1,41876E-13	-0,606779002	4,5553E-07	2,41847E-05
ENSG00000128000	ZNF780B	0,44664503	4,90773E-16	1,51549E-13	0,363821672	1,98534E-09	3,30112E-07
ENSG00000153551	CMTM7	-0,466477212	5,29089E-16	1,55609E-13	-0,380577685	1,98038E-09	3,30112E-07
ENSG00000157613	CREB3L1	-0,952133308	6,19974E-16	1,70561E-13	-0,582184526	7,3057E-06	0,00018736
ENSG00000100997	ABHD12	-0,437859251	7,54868E-16	1,96929E-13	-0,274645324	4,3255E-06	0,000125622

Supplementary Table 5. Top 50 up- and downregulated genes in Type2 / Ctrl, ordered by *padj* value.

ensembl	hgnc_symbol	log2FoldChange_type2	pvalue_type2	padj_type2	log2FoldChange_type1	pvalue_type1	padj_type1
ENSG00000121871	SLITRK3	2,275486085	1,18182E-28	1,78821E-24	2,863280258	1,58232E-53	2,39421E-49
ENSG00000117114	ADGRL2	1,036819058	1,30184E-22	9,84908E-19	0,952064679	4,18797E-23	1,26736E-19
ENSG00000154118	JPH3	2,327879847	8,31723E-22	4,19493E-18	2,082907384	3,40327E-21	4,68135E-18
ENSG00000181409	AATK	1,497059407	3,07538E-19	9,30671E-16	1,321341694	3,29005E-18	2,16442E-15
ENSG00000147588	PMP2	2,386053153	1,05195E-18	2,65284E-15	2,014741804	3,20725E-16	1,05498E-13
ENSG00000179915	NRXN1	2,003575781	1,95443E-17	4,22464E-14	1,666826959	7,85937E-15	1,46815E-12
ENSG00000158887	MPZ	2,079029259	2,73317E-17	5,16944E-14	1,78229236	1,45842E-15	3,39497E-13
ENSG00000175161	CADM2	2,513266952	1,39074E-16	2,10432E-13	2,244629142	6,05133E-16	1,69561E-13
ENSG00000078401	EDN1	1,552968971	1,67229E-16	2,17875E-13	1,299436335	3,03758E-14	4,4623E-12
ENSG00000046653	GPM6B	1,430186038	1,72791E-16	2,17875E-13	1,128770773	8,00488E-13	6,96103E-11
ENSG00000151892	GFRA1	1,450410084	1,98284E-14	2,14302E-11	1,643902733	1,20011E-21	2,26985E-18
ENSG00000092758	COL9A3	1,252118273	4,11425E-14	3,89079E-11	1,319956721	1,75118E-18	1,32485E-15
ENSG00000163873	GRIK3	2,060335676	4,41652E-14	3,93096E-11	1,867987319	5,48245E-14	7,27675E-12
ENSG00000206579	XKR4	1,895626787	6,12371E-14	5,14766E-11	1,776279702	1,05766E-14	1,90518E-12
ENSG00000172005	MAL	1,453983981	1,80193E-13	1,35331E-10	1,415481586	4,07464E-15	8,11228E-13
ENSG00000165238	WNK2	1,12268634	1,87824E-13	1,35331E-10	1,052272561	3,01498E-14	4,4623E-12
ENSG00000186766	FOXI2	1,455368069	3,37187E-13	2,31908E-10	1,675032858	3,96349E-20	4,6132E-17
ENSG00000227467	LINC01537	1,089033648	4,37877E-13	2,88066E-10	0,88339857	2,00108E-10	6,37438E-09
ENSG00000197261	C6orf141	1,233097125	1,33577E-12	8,08459E-10	1,271706234	9,24343E-16	2,29283E-13
ENSG00000160307	S100B	2,090534678	1,67988E-12	9,77623E-10	1,576153018	5,04782E-09	9,64376E-08
ENSG00000120729	MYOT	1,411798685	1,82143E-12	1,02074E-09	1,068294004	5,93425E-09	1,09501E-07
ENSG00000065361	ERBB3	1,577941088	2,7213E-12	1,47057E-09	1,400068551	8,51616E-12	4,84428E-10
ENSG00000047617	ANO2	1,070224219	3,24643E-12	1,6754E-09	0,749945214	9,8747E-08	1,16005E-06
ENSG00000114200	BCHE	1,53033441	6,90744E-12	3,07401E-09	1,779458547	1,46733E-18	1,16853E-15
ENSG00000213397	HAUS7	1,597101075	7,28707E-12	3,1503E-09	1,936793497	5,40138E-20	5,83773E-17
ENSG00000121966	CXCR4	-1,832988925	4,95067E-20	1,87271E-16	-1,683375246	1,52445E-20	1,92221E-17
ENSG00000198851	CD3E	-1,633277359	8,76316E-17	1,47328E-13	-1,421566161	9,17828E-16	2,29283E-13
ENSG00000136738	STAM	-0,337224827	2,28312E-16	2,65738E-13	-0,315698646	2,6447E-17	1,37989E-14
ENSG00000271503	CCL5	-1,492627826	2,70913E-14	2,73279E-11	-1,408642596	2,26693E-15	4,97114E-13
ENSG00000090104	RGS1	-2,495009269	1,74557E-13	1,35331E-10	-2,153023474	2,26935E-12	1,69988E-10
ENSG00000160679	CHTOP	-0,236484059	1,1572E-12	7,29568E-10	-0,157646618	1,7052E-07	1,82729E-06
ENSG00000136895	GARNL3	0,474305326	3,32178E-12	1,6754E-09	0,387846087	3,66862E-10	1,07162E-08
ENSG00000154065	ANKRD29	0,477558541	3,47853E-12	1,69786E-09	0,541507288	3,47744E-18	2,19238E-15
ENSG00000153563	CD8A	-1,589214472	4,18958E-12	1,97717E-09	-1,566061413	4,88449E-14	6,71884E-12
ENSG00000204335	SP5	-1,51309355	4,3121E-12	1,97717E-09	-1,642372928	1,46689E-16	5,42183E-14
ENSG00000218336	TENM3	-1,242473195	9,46506E-12	3,8707E-09	-1,506435434	8,54373E-20	8,61835E-17
ENSG00000172216	CEBPB	-0,911656986	1,65414E-11	5,82066E-09	-1,212403401	5,6826E-23	1,43306E-19
ENSG00000109220	CHIC2	-0,41976378	2,18263E-11	7,50575E-09	-0,487311537	1,19834E-17	6,71559E-15
ENSG00000143320	CRABP2	-1,354216698	4,02474E-11	1,26871E-08	-1,413893375	2,82548E-14	4,27523E-12
ENSG00000258754	LINC01579	-1,747069332	4,60249E-11	1,3928E-08	-1,975353374	2,30219E-16	7,91692E-14
ENSG00000167378	IRGQ	0,360958571	5,06722E-11	1,50338E-08	0,414499309	8,87278E-17	3,68468E-14
ENSG00000057657	PRDM1	-1,211135913	5,56803E-11	1,58962E-08	-1,028807682	7,99245E-10	2,07433E-08
ENSG00000145868	FBXO38	-0,259952758	7,35424E-11	2,02322E-08	-0,276016069	2,55464E-14	3,98498E-12
ENSG00000143079	CTTNBP2NL	-0,390721017	1,18085E-10	2,97791E-08	-0,431689373	4,31059E-15	8,36198E-13
ENSG00000173926	MARCH3	-1,147967968	1,2266E-10	2,9935E-08	-1,235705405	2,15892E-14	3,47517E-12
ENSG00000172667	ZMAT3	-0,545706534	3,89081E-10	8,78685E-08	-0,658947703	8,0451E-17	3,47801E-14
ENSG00000128340	RAC2	-0,965296419	4,91383E-10	1,0934E-07	-0,882010141	3,33897E-10	9,88687E-09
ENSG00000185753	CXorf38	-0,298455795	5,03493E-10	1,09538E-07	-0,182307836	2,72507E-05	0,000134323
ENSG00000122042	UBL3	-0,355610539	5,0675E-10	1,09538E-07	-0,51032051	8,36932E-23	1,80909E-19
ENSG00000169252	ADRB2	-0,864381676	5,82171E-10	1,24068E-07	-0,810616871	1,44813E-10	4,91292E-09
